# TCRconv: predicting recognition between T cell receptors and epitopes using contextualized motifs

**DOI:** 10.1093/bioinformatics/btac788

**Published:** 2022-12-07

**Authors:** Emmi Jokinen, Alexandru Dumitrescu, Jani Huuhtanen, Vladimir Gligorijević, Satu Mustjoki, Richard Bonneau, Markus Heinonen, Harri Lähdesmäki

**Affiliations:** Department of Computer Science, Aalto University, Espoo 02150, Finland; Department of Computer Science, Aalto University, Espoo 02150, Finland; Helsinki Institute of Life Science, University of Helsinki, Helsinki 00014, Finland; Department of Clinical Chemistry and Hematology, Translational Immunology Research Program, University of Helsinki, Helsinki 00290, Finland; Hematology Research Unit Helsinki, Helsinki University Hospital Comprehensive Cancer Center, Helsinki 00290, Finland; Center for Computational Biology (CCB), Flatiron Institute, Simons Foundation, New York, NY 10010, USA; Prescient Design, Genentech, New York, NY, USA; Department of Clinical Chemistry and Hematology, Translational Immunology Research Program, University of Helsinki, Helsinki 00290, Finland; Hematology Research Unit Helsinki, Helsinki University Hospital Comprehensive Cancer Center, Helsinki 00290, Finland; iCAN Digital Precision Cancer Medicine Flagship, Helsinki, Finland; Center for Computational Biology (CCB), Flatiron Institute, Simons Foundation, New York, NY 10010, USA; Prescient Design, Genentech, New York, NY, USA; Center for Data Science, New York University, New York, NY 10011, USA; Department of Computer Science, New York University, Courant Institute of Mathematical Sciences, New York, NY 10012, USA; Department of Computer Science, Aalto University, Espoo 02150, Finland; Department of Computer Science, Aalto University, Espoo 02150, Finland

## Abstract

**Motivation:**

T cells use T cell receptors (TCRs) to recognize small parts of antigens, called epitopes, presented by major histocompatibility complexes. Once an epitope is recognized, an immune response is initiated and T cell activation and proliferation by clonal expansion begin. Clonal populations of T cells with identical TCRs can remain in the body for years, thus forming immunological memory and potentially mappable immunological signatures, which could have implications in clinical applications including infectious diseases, autoimmunity and tumor immunology.

**Results:**

We introduce TCRconv, a deep learning model for predicting recognition between TCRs and epitopes. TCRconv uses a deep protein language model and convolutions to extract contextualized motifs and provides state-of-the-art TCR-epitope prediction accuracy. Using TCR repertoires from COVID-19 patients, we demonstrate that TCRconv can provide insight into T cell dynamics and phenotypes during the disease.

**Availability and implementation:**

TCRconv is available at https://github.com/emmijokinen/tcrconv.

**Supplementary information:**

Supplementary data are available at *Bioinformatics* online.

## 1 Introduction

T cell receptors (TCRs) form diverse repertoires through V(D)J recombination, which allows T cells to recognize a large variety of antigens. Short peptide sequences from the antigens, called epitopes, are presented to T cells via major histocompatibility complex (MHC) molecules, and successful recognition of an epitope–MHC complex by a TCR results in T cell activation and immune response against the antigen. Discovering epitope-specific TCRs holds the potential to provide clinically relevant insights into TCR repertoires in fields ranging from vaccine design and diagnostics to immunotherapy biomarker identification.

Latest high-throughput sequencing technologies have enabled profiling large quantities of TCR sequences. Concurrently, several methods have been proposed for predicting TCR-epitope recognition that include Gaussian processes (TCRGP by Jokinen *et al.*, 2021), deep learning methods [DeepTCR by Sidhom *et al.* (2021), and ERGO-II by Springer *et al.* (2021)], gradient boosting decision trees [SETE by Tong *et al.* (2020)] and TCRdist by Dash *et al.* (2017). Apart from ERGO-II all these methods use epitopes as class information to predict if a TCR would recognize one of the predetermined epitopes, which may still be more reliable with the limited amount of epitope-specific TCR data available. TCRGP is a Gaussian process-based classifier that can utilize complementarity determining region 3 (CDR3) or additionally any other CDRs from either α- or β-chain or both, depending on what information is available. DeepTCR uses convolutional neural networks (CNNs) with trainable embedding layers for the CDR3 and V/D/J genes. SETE on the other hand computes the PCA of 3-mer occurrences in CDR3 regions and uses gradient boosting decision trees to classify CDR3 sequences. TCRdist uses a BLOSUM62-based distance measure between selected CDRs to determine if a new test TCR is closer to TCRs specific to a certain epitope or to some non-specific control TCRs. ERGO-II learns long short-term memory encodings for both CDR3s and epitopes (or autoencoder embeddings for CDR3s) and uses both the TCR and the epitope as inputs to predict if a given TCR and epitope bind.

Previous work has shown that while the CDR3 is crucial for the prediction, it is beneficial to utilize also other TCR regions as well as the paired TCRαβ sequences (Dash *et al.*, 2017; Jokinen *et al.*, 2021; Sidhom *et al.*, 2021; Springer *et al.*, 2021). We focus on major open questions in TCR-epitope prediction: how to (i) utilize efficiently all TCR regions that determine epitope-specificity, (ii) handle TCR cross-reactivity and (iii) use TCR-epitope prediction methods for unsupervised analysis of TCR-repertoires.

**Fig. 1.**
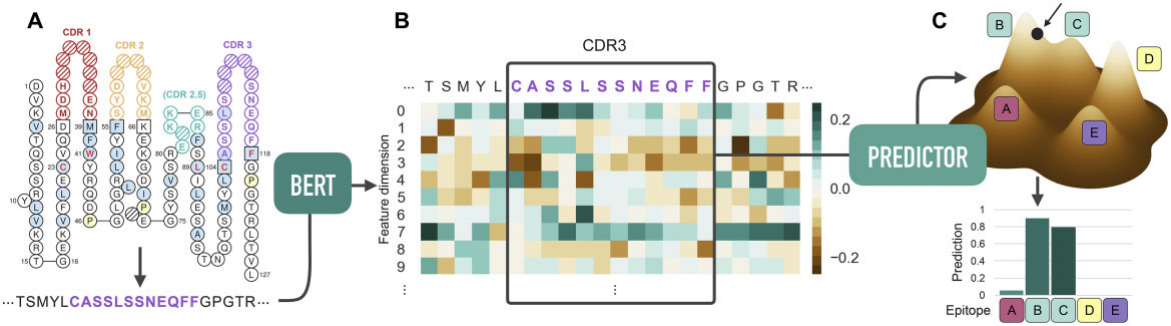
TCRconv pipeline. (**A**) The TCR sequence determined by V(D)J recombination contains the complementarity-determining regions. TCRα and/or TCRβ sequences can be used, here TCRβ is shown. (**B**) ProtBERT embedding is created for each TCR sequence and the CDR3 embedding, transfused with information from its context, is extracted. (**C**) The multilabel predictor produces simultaneously separate predictions for each epitope

Here, we present TCRconv, a CNN that utilizes rich contextualized transformer embeddings of TCRs to predict epitope recognition (see Fig. 1). Unlike the previous methods, TCRconv models TCR specificity with a multilabel predictor that naturally accounts for TCR cross-reactivity. Transformer-based language models, such as BERT (Bidirectional Encoder Representations from Transformers), have been adapted for proteins and can capture protein folding as well as learn useful representations of binding sites and complex biophysical properties (Vig *et al.*, 2020). They have been successfully used in various tasks, including protein family and protein interaction prediction (Nambiar *et al.*, 2020) and protein-specific drug generation (Grechishnikova, 2021), making them a plausible candidate for TCR-epitope prediction. We utilize the transformer model protBERT (Elnaggar *et al.*, 2020), which transfers information from the complete TCR sequence to the CDR3 embedding from which the convolutional networks then extract and utilize contextualized motifs.

## 2 Materials and methods

Lets assume that we have *N* TCRs that are represented by their amino acid sequences $a_{n}=[a_{n,1},a_{n,2},\ldots ,a_{n,L_{n}}]$, where $a_{n,i}\in \{A,R,N,\ldots ,V\}$ represents one of the 20 naturally occurring amino acids and *L_n_* is the length of the *n*^th^ TCR. Each of the *N* TCRs is paired with a multihot encoding. For TCR $a_{n}$ the encoding $y_{n}=[y_{n,1},y_{n,2},\ldots ,y_{n,C}]$ defines which of the *C* epitopes the TCR recognizes:

$$y_{n,c}=\{\begin{array}{cc} 1, & \text{if TCR}\;a_{n}\;\text{recognizes epitope}\;c\\ 0, & \text{otherwise}. \end{array}.$$

The labeled data are denoted collectively as $D={\left\{(a_{n},y_{n})\right\}}_{n=1}^{N}$.

### 2.1 Data

For training and testing our model, we have constructed three datasets of human TCR sequences from the data available in the VDJdb database by Bagaev *et al.* (2020) (vdjdb.cdr3.net). VDJdb gives confidence scores from 0 to 3 for each of its entries (see Supplementary Section S1). For a comprehensive dataset VDJdbβ-large, we selected TCRβs with all confidence scores and with at least 50 unique TCRβs for each epitope (a TCR is considered as unique if the combination of its CDR3 and V- and J-genes is unique). This resulted in a dataset with 51 distinct epitopes and 30 503 unique TCRβs. For a high-quality dataset VDJdbβ-small we chose TCRβs with at least a confidence score of 1 and at least 40 unique TCRs per epitope, which resulted in 1977 unique TCRs specific for 21 epitopes. Finally, dataset VDJdbαβ-large consists of paired TCRαβ sequences with all confidence scores and at least 50 unique TCRαβs per epitope, resulting in total 20 200 unique TCRs and 18 epitopes. Supplementary Table S1 summarizes these datasets and the cross-reactivities of the TCRs are visualized in Supplementary Figure S1. As the requirements for the datasets overlap, so do the datasets: e.g. VDJdbβ-large contains the complete VDJdbβ-small dataset.

All presented model evaluations are conducted using a stratified 10-fold cross-validation, where TCRs specific to each epitope are distributed to the folds as evenly as possible. As our dataset only consists of unique TCRs, the same TCR can never be both in training and test folds. To illustrate the difficulty of predicting the epitope specificity of TCRs with the chosen data, we visualized the CDR3 edit-distances from each TCR specific to an epitope to TCRs with the same specificity and to TCRs with other specificity (Supplementary Fig. S3). For example, in VDJdbβ-large dataset, for 37 of the 51 epitopes the nearest TCRs are more often specific to another epitope than to the same epitope. Further, Supplementary Figure S4 shows the edit distances from TCRs specific to the chosen epitope to all TCRs with the same and other specificity, illustrating that a simple measure such as an edit-distance is not sufficient for assessing if TCRs share the same epitope-specificity. The corresponding edit-distance plots for VDJdbβ-small are shown in Supplementary Figure S5. The difficulty of classifying epitope-specific TCRs is further highlighted by the UMAP visualizations of the BERT embeddings for TCRs in Supplementary Figures S6 and S7. With most epitopes the TCRs recognizing them are scattered with no defined clusters. With a few epitopes, such as IAV M_GILGFVFTL_, there are several small clusters, but a large part of the epitope-specific TCRs are still scattered.

### 2.2 TCR embeddings

For constructing the TCR embeddings, we used protBERT (Elnaggar *et al.*, 2020) which is trained on 216 million UniRef100 sequences. The model was trained with a token-prediction task and during the training phase 15% of the tokens (amino acids) in the sequences were replaced by a MASK token. The model contains 16 attention heads in each multi-head attention block on 30 layers, with 420 million parameters in total. The embedding dimension for each amino acid is 1024. The embeddings are contextualized, which means that the representation of an amino acid depends on the TCR sequence surrounding it and thus contains information from its context.

Given an amino acid sequence $a_{n}=[a_{n,1},a_{n,2},\ldots ,a_{n,L_{n}}]$ of a TCR, protBERT computes a corresponding embeddings ${\tilde{X}}_{n}=[x_{n,1},x_{n,2},\ldots ,x_{n,L_{n}}]$, where $x_{n,i}\in R^{1024}$. The length of the TCR sequences, *L_n_*, defined by V-genes, CDR3 sequences and J-genes, varies roughly between 100 and 140 amino acids. We extract the part of the embedding corresponding to the CDR3, $X_{n}=[x_{n,r_{n}},x_{n,r_{n}+1},\ldots ,x_{n,r_{n}+l_{n}-1}]$. The CDR3 embedding provides a more compact presentation of the TCR that is still transfused with information from its context.

We also experimented with a protBERT model fine-tuned with TCR sequences, two different ELMO (Embeddings from Language Models) architectures, and one-hot encodings for the CDR3 and TCR sequences, but the original protBERT model had the best performance. See Supplementary Section S2 for details.

### 2.3 CNN predictor

**Fig. 2.**
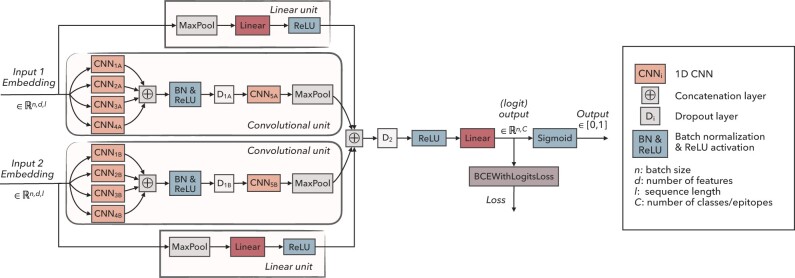
TCRconv multilabel predictor. TCRconv can utilize one or two inputs, i.e. embedding for TCRβ and/or TCRα. Each sequence embedding goes through a convolutional and a linear unit in parallel. The outputs from these units (for each TCR chain) are concatenated and go through a final linear layer. During training, weighted binary cross-entropy with logits loss (BCEWithLogitsLoss which includes a sigmoid function) is utilized, and the predictions are squashed between 0 and 1 by a sigmoid layer, separately for each epitope

Once the protBERT embeddings are computed for the TCRs, our training data consists of pairs $\left(X_{n},y_{n}\right)$ that are used for training our predictor network $f(X_{n};W)={\hat{y}}_{n}$ with parameters *W*. Our multilabel classifier consists of a parallel convolutional unit and a simple linear unit for each TCR chain and was motivated by the CNN classifier presented by Gligorijević *et al.* (2021) (see Fig. 2). The convolutional unit consists of parallel convolutional layers with varying kernel sizes (5, 9, 15 and 21, with 120, 100, 80 and 60 filters, respectively) that can capture different length motifs. The outputs from these layers are concatenated and fed through batch normalization, rectified linear unit (ReLU) activation, and a dropout layer with 0.1 dropping probability. Those are followed by another convolutional layer (kernel size 3, 60 filters) that can extract higher level features based on the outputs from the previous convolutional layers. Finally, max pooling is performed over the sequence lengths, which provides fixed sized outputs regardless of the sequences’ lengths. As our input embeddings are contextualized, the convolutional unit extracts contextualized motifs where the surroundings of the motif even outside the CDR3 also affect how it is perceived. The linear unit can more flexibly utilize the expressive features of the BERT embeddings. It consists of a max pooling layer, a linear layer, and a ReLU activation. The outputs of the convolutional and linear units are concatenated and put through a dropout layer with dropping probability 0.1, batch normalization and ReLU. The final linear layer gives predictions simultaneously for each class that are separately squashed between 0 and 1 by a sigmoid layer.

To optimize the parameters *W* of the network, we minimize the binary cross-entropy (BCE) with logits loss between the true labels $y_{n}$ and predicted labels ${\hat{y}}_{n}$, simultaneously for all epitopes. For TCR n∈{1,…,N} and label c∈{1,…,C}, the loss is defined as

(1) $$\begin{array}{c} \ell ({\hat{y}}_{n,c},y_{n,c})=-[p_{c}\cdot y_{n,c}\cdot \;\text{log}\;\sigma ({\hat{y}}_{n,c})\\ +(1-y_{n,c})\cdot \;\text{log}(1-\sigma ({\hat{y}}_{n,c}))], \end{array}$$

where $p_{c}=N/\left(m_{c}\cdot C\right)$ is the weight for positive samples from class *c* of size *m_c_*, and $\sigma (y)=1/\left(1+e^{-y}\right)$ is the sigmoid function. Loss over all TCRs and labels is then defined as

(2) $$\text{Loss}=\frac{1}{NC}\sum_{c=1}^{C}\sum_{n=1}^{N}\ell ({\hat{y}}_{n,c},y_{n,c}).$$

This multilabel formulation and usage of BCE loss allows us to account for also cross-reacting TCRs that can recognize multiple epitopes.

For training the models, we use stochastic weight averaging (SWA) with learning rate scheduling (Izmailov *et al.*, 2018). The models are first trained for 2500 iterations (mini-batches) without weight averaging but with cosine annealing for the learning rates, so that the learning rates gradually decrease. After that, the training is continued for another 500 iterations with SWA on every iteration and again a decreasing learning rate is used. The learning rate is 0.0002, except for the linear unit, for which the learning rate is set to 0.01.

### 2.4 Comparison to other methods

We compared TCRconv to recently published methods for predicting TCR epitope-specificities, TCRGP (Jokinen *et al.*, 2021), DeepTCR (Sidhom *et al.*, 2021), SETE (Tong *et al.*, 2020), TCRdist (Dash *et al.*, 2017) and ERGO-II (Springer *et al.*, 2021). While our TCRconv can be trained simultaneously for all epitopes using the multi-hot labels, with TCRGP, DeepTCR, SETE, and TCRdist we trained separate binary classifiers for each epitope, so that TCRs known to recognize the epitope in question are considered as positive data points and TCRs specific to other epitopes are considered as negative data points. DeepTCR and SETE have options for multiclass classification, but they do not provide support for cross-reactive TCRs that our data contains. Therefore, they would have had a disadvantage if trained as multiclass classifiers as they then would have operated with either conflicted or missing class labels. ERGO-II takes TCR-epitope pairs as inputs and predicts if the pairs bind. Non-binding TCR-epitope pairs then need to be generated for its training.

We compared the above methods on our two TCRβ datasets, VDJdbβ-large and VDJdbβ-small, using stratified 10-fold cross-validation. The folds used in the cross-validation were the same for each of these methods. As suggested by the authors, with DeepTCR 25% and with ERGO-II 20% of the training data were used as validation data for determining early stopping when training the classifiers. With ERGO-II, we used all the binding TCR-epitope pairs in our data, but additionally sampled five times more non-binding data, replicating their training procedure. Therefore, when with TCRconv e.g. TCR CASLSGRAPQHF, TRBV27*01 occurs once in VDJdbβ-small with a multi-hot encoding indicating that it can recognize epitopes GTSGPIINR and GTSGPIVNR, with ERGO-II it is repeated 12 times, twice in a positive pair with both GTSGPIINR and GTSGPIVNR, and 10 times in negative pairs formed by randomly selecting 10 of the other 19 epitopes in the dataset.

### 2.5 TCR diversity

To estimate the diversity of *N* TCRs specific to a certain epitope, we utilized a diversity measure similar to measures used in previous studies (Dash *et al.*, 2017; Jokinen *et al.*, 2021). These measures are based on Simpson’s diversity index, but due to the large variety of TCRs, they measure similarities between TCRs instead of exact matches. Here, the similarity between TCRs *n* and *j* is computed based on the used embeddings ${\bar{X}}_{n}$ and ${\bar{X}}_{j}$ that have been *aligned* based on IMGT numbering:

(3) $$\text{diversity}={\left(\frac{\sum_{n=1}^{N-1}\sum_{k=n+1}^{N}\;\text{exp}\;\left(-\frac{||{\bar{X}}_{n}-{\bar{X}}_{j}|{|}_{\text{Fro}}^{2}}{2\;s.d.^{2}}\right)}{\frac{1}{2}(N-1)N}\right)}^{-1},$$

where $||X|{|}_{\text{Fro}}$ denotes the Frobenius norm of **X**, and s.d. is set to 10.4 (maximum feature-wise standard deviations multiplied by the median sequence length 14).

## 3 Results

### 3.1 Comparison to other methods

**Fig. 3.**
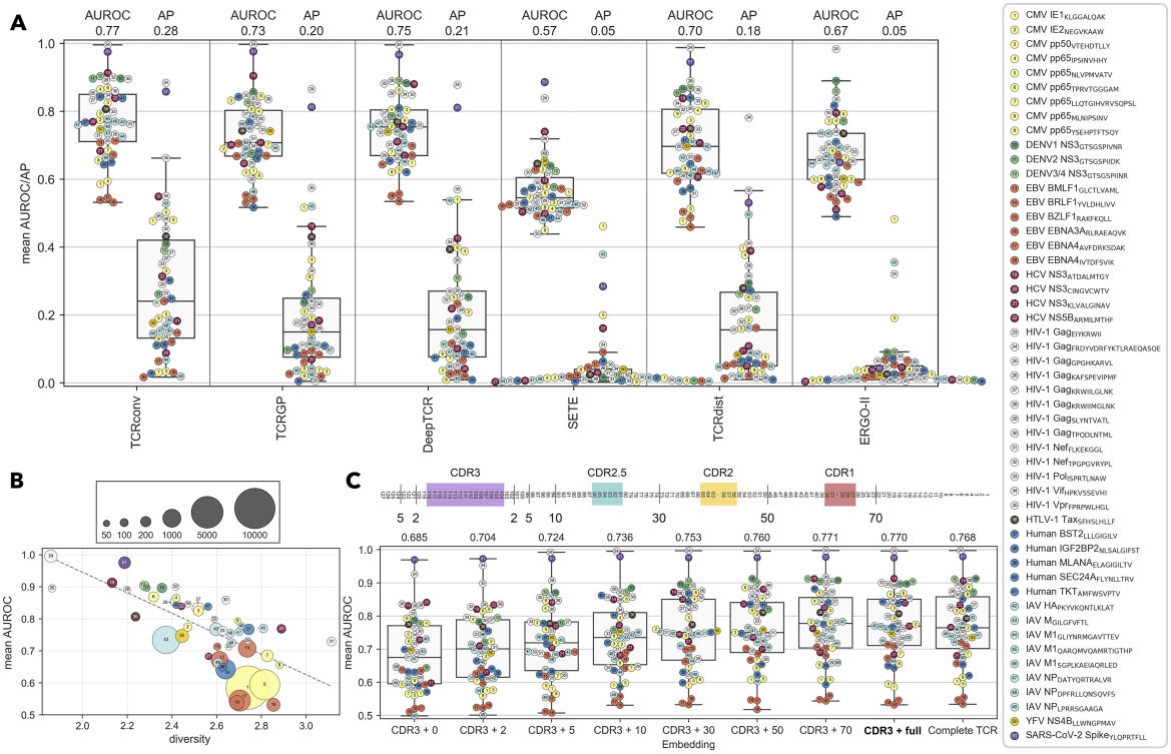
TCRconv evaluation. Results are obtained using stratified 10-fold cross-validation on VDJdbβ-large dataset. (**A**) Comparing TCRconv to other methods using average AUROC and AP scores. (**B**) The AUROC scores for TCRconv predictions correlate negatively with the diversity of the epitope specific TCRs (Pearson correlation −0.72). (**C**) Increasing the embedding context size increases the predictive AUROC score. The schematics on top show the approximate sections included in different context sizes. CDR3 + X refers to CDR3 embeddings with context size X and complete TCR to embeddings for complete TCRs without extracting only the CDR3 parts. TCRconv uses CDR3 + full (bolded)

We first compared the prediction accuracies of TCRconv and previous methods, TCRGP, deepTCR, TCRdist, SETE and ERGO-II, on the two epitope-specific datasets: a comprehensive VDJdbβ-large consisting of data with all confidence levels, and a smaller high-quality VDJdbβ-small (see Section 2.1 and Supplementary Table S1). Prediction accuracies are quantified using the average precision (AP), which accounts for class imbalances, and the area under the receiver operating characteristic curve (AUROC). TCRconv achieves the highest AP and AUROC scores on VDJdbβ-large (33% and 3% improvement to the second best DeepTCR) (Fig. 3A). High AP scores are essential as minimizing false positive predictions with large TCR repertoires and small TCR clones (Qi *et al.*, 2014) is crucial. Overall, all methods performed better on the higher confidence, albeit smaller, VDJdbβ-small dataset (Supplementary Fig. S8 and Table S2).

### 3.2 TCR cross-reactivity and diversity

As TCRs can be cross-reactive (Supplementary Fig. S1), TCRconv benefits from using a single multilabel predictor that can predict a TCR to recognize several epitopes. Whereas, to account for cross-reactivity with previous binary (Dash *et al.*, 2017; Jokinen *et al.*, 2021) and multiclass classifiers (Sidhom *et al.*, 2021; Tong *et al.*, 2020), a large set of separate (one-vs-all) classifiers had to be trained, one for each epitope. TCRconv performs well also with cross-reacting TCRs (Supplementary Fig. S2). Further, consistent with previous results (Dash *et al.*, 2017; Jokinen *et al.*, 2021), we confirmed that prediction accuracy across epitopes correlates negatively with the diversity of the TCRs recognizing these epitopes (Fig. 3B, Supplementary Fig. S9A).

### 3.3 Effect of the CDR3 context size and αβ-chain usage

CDR3 is essential in epitope recognition, but structural (Glanville *et al.*, 2017) and computational (Dash *et al.*, 2017; Jokinen *et al.*, 2021) evidence suggests that CDR1 and CDR2, which mainly contact the MHC, may also interact with the epitope and aid the prediction. We next evaluated how much of the TCR sequence around the CDR3 should be used as context when computing the protBERT embedding. The prediction AUROC score improves gradually from 0.68 to 0.77 when the context size is increased from no context to full context (i.e. the full-length TCRβ sequence, or VDJ-sequence) on VDJdbβ-large (Fig. 3C), indicating that protBERT successfully conveys relevant information from the context to the CDR3 embedding. Using the CDR3 with full context corresponds to using both V- and J-genes in addition to the CDR3, since the region before the CDR3 is encoded by the V-gene and the region after CDR3 by the J-gene. With both datasets the AUROC and AP scores improve or remain the same when using context as far as before CDR1 (Fig. 3C, Supplementary Fig. S9B). Remarkably, the entire TCR embedding is not needed, but using the CDR3 embedding with full context provides similar or slightly better results.

**Fig. 4.**
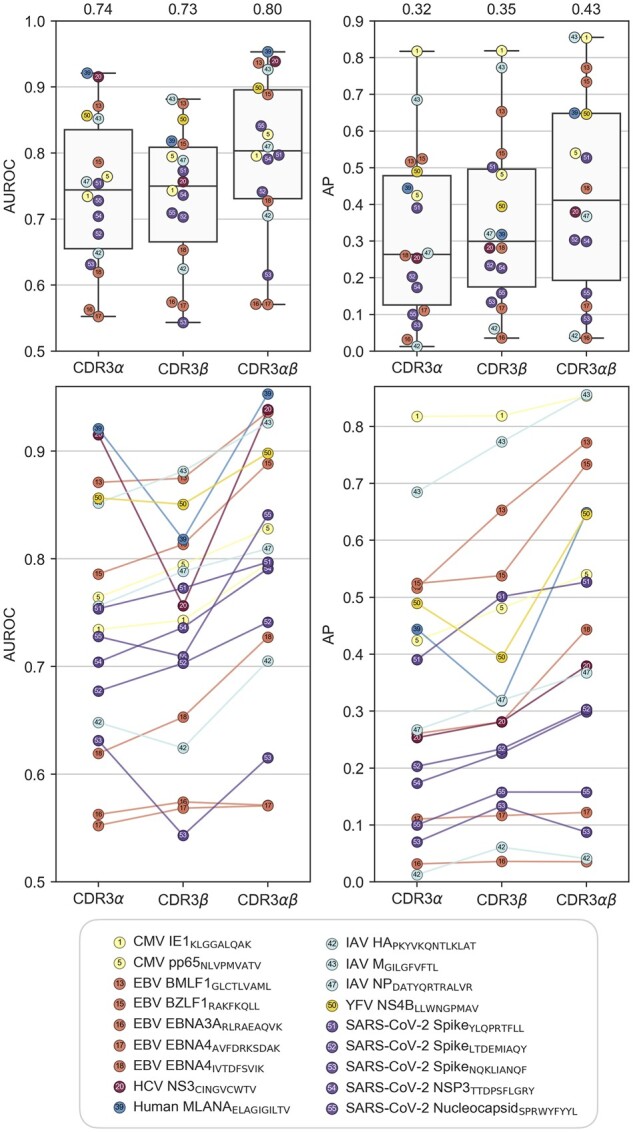
TCRconv performs best when using both α- and β-chains. Results are obtained on VDJdbαβ-large dataset in terms of average AUROC and AP scores over stratified 10-fold cross-validation. Each circle corresponds to TCRs specific to one epitope as described in the legend. Above boxplots show the distribution of the prediction accuracies when the TCRconv model is trained using embeddings for CDR3α, CDR3β or both (always with the full context, meaning that an embedding is first computed for the complete TCR determined by the CDR3, and V- and J-genes, and then the part corresponding to the CDR3 is extracted). Mean metrics are shown on top of each boxplot. Below the circles from the three models are connected by lines, illustrating how for most epitopes the best results are obtained when using both chains and that using β-chains is better than using α-chains, although there are exceptions

TCR is a dimeric molecule that consists of α and β chains. While TCRβ is more often in close proximity to an epitope than TCRα, usually both chains are within 5 Å of the epitope (Glanville *et al.*, 2017). We studied the effect of TCRα and TCRβ on TCR-epitope prediction on VDJdbαβ-large dataset of paired TCRαβ sequences (Supplementary Table S1). We found substantial performance improvement from using both chains over either chain individually (Fig. 4). Further, when using either chain individually, it varies which chain provides the best accuracy. With most epitopes, the accuracy is better when using the β-chain, but with some epitopes using the α-chain is clearly more beneficial. When using both chains, the increase in accuracy when compared to either chain individually is more systematic.

With complex models such as protBERT and multiple convolutional layers, each with several filters, it can be challenging to identify any clear motifs that would be important for the predictions. In Supplementary Section S3, we discuss how saliency maps can be used for this purpose.

### 3.4 The effect of HLA-type

As the TCR regions outside the CDR3 do not often interact with an epitope but may interact with the MHC molecule presenting the epitope, the HLA-type of the MHC can affect the recognition between the TCR and the epitope. Therefore, utilizing these regions could introduce a bias in the epitope-specificity prediction. Although the available TCR-epitope-MHC complexes in VDJdb contain various HLA-types, most of the data are restricted to HLA-A*02 and almost all the epitopes are presented by a single HLA-group (see Supplementary Fig. S10A). This makes it difficult to model or even assess how different HLA-types affect the TCRs’ ability to bind certain epitopes. To ensure that our multilabel predictor is predicting a TCR’s ability to bind to an epitope and not to the HLA presenting it, we examined how much the results differ between different HLA-genes as well as between TCRconv models trained on data restricted by any HLA type or only with HLA-A*02 restricted data. The number of epitopes restricted by each HLA-gene is limited and the prediction accuracy varies considerably between epitopes, but Supplementary Figure S10B indicates that differences between HLA-genes are modest (AUROC across genes varies from 0.743 to 0.810, while AUROC across epitopes varies from 0.532 to 0.996). Further, Supplementary Figure S10C shows that the accuracy is similar when TCRconv is trained on all data or only with HLA-A*02 restricted epitopes. These results suggest that TCRconv predicts TCR’s ability to bind epitopes and not the HLAs.

### 3.5 Dynamics and phenotypes of SARS-CoV-2-specific TCRs in COVID-19

Finally, we demonstrate how to utilize TCRconv in repertoire data analysis to track T cell dynamics (Snyder *et al.*, 2020) during coronavirus disease 2019 (COVID-19) and to reveal the phenotypes of severe acute respiratory syndrome coronavirus 2 (SARS-CoV-2) specific T cells in moderate and severe COVID-19. We first trained a TCRconv model specifically for SARS-CoV-2 epitopes using ImmuneCODE (Nolan *et al.*, 2020) and VDJdbβ-large data.

#### 3.5.1 TCRconv model for SARS-CoV-2 epitopes

**Fig. 5.**
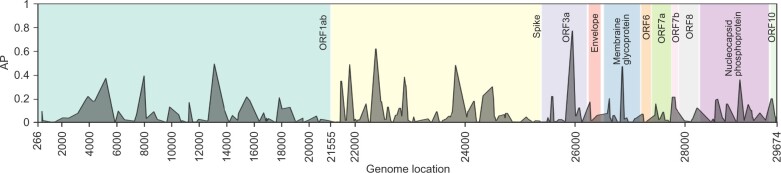
TCRconv prediction performance for SARS-CoV-2 epitopes by the peptides’ genome location in terms of AP scores. The ORF1ab region has been compressed

For training TCRconv models for SARS-CoV-2 epitopes, we utilized ImmuneCODE MIRA-data of TCRs specific to MHC-I restricted peptides. To fully exploit the data, we did the following preprocessing with three options for the TCR sequences: (i) If the V- and J-genes and their alleles could be determined from the nucleotide sequence (length 29 nucleotides), we used the exact TCR amino acid sequence determined by the CDR3β, V- and J-genes. (ii) If a V- or J- gene could be determined but not its allele, we set the allele to 01 and used it for constructing the amino acid sequence. (iii) If a gene could not be determined, we utilized a partial amino acid sequence that we could uniquely determine based on the nucleotide sequence. BERT embeddings were computed for these TCRβ sequences and the parts of the embeddings corresponding to the CDR3s were extracted and used with the CNN predictor. TCR uniqueness was determined by these longest amino acid sequences that we could obtain. We selected 139 099 unique TCRβs specific to 188 peptide groups with at least 50 unique TCRβs specific to them (see Supplementary Table S3) and used stratified 10-fold cross-validation with these TCRs to evaluate TCRconv on this data. The performance in terms of AP scores for the TCRs specific to each peptide group is shown by the peptide group’s genomic location in Figure 5, and Supplementary Figure S11A additionally shows the AUROC scores and diversity of these TCRs. The mean AUROC and AP scores are shown in Supplementary Figure S11B. We then selected the twenty peptide groups that performed best in terms of weighted mean of AUROC and AP scores (both scores were scaled into range [0,1]) and used the corresponding TCRs together with VDJdbβ-large dataset to construct the final predictor (performance using stratified 10-fold cross-validation is shown in Supplementary Fig. S11C).

#### 3.5.2 Dynamics of SARS-CoV-2-specific TCRs

To track the T cell dynamics during COVID-19, we selected 493 COVID-19 patients from ImmuneCODE repertoires and 110 healthy controls from (Emerson *et al.*, 2017). We utilized TCR repertoires from ImmuneCODE that contain at least 250 000 TCRs and the number of days between diagnosing the patient and collecting the sample is reported. For the control repertoires, we also required at least 250 000 TCRs and only selected subjects with age at least 18 years (which is the age of the youngest subject in ImmunoCode data). The data are described in Supplementary Table S4. The sequences were preprocessed in the same way as the ImmuneCODE MIRA-data and each sample was downsampled to 250 000 TCRs. Using the TCRconv model for SARS-CoV-2 epitopes, we predicted the specificity for each of the approx. 150M T cells within these repertoires. We chose a threshold separately for each epitope that corresponds to false positive rate of 0.001. With thresholds this strict we are not likely to find all TCRs specific to the selected epitopes but have a high confidence in that the TCRs predicted to recognize these epitopes are true positives. We computed the frequency of SARS-CoV-2-specific TCRs in each repertoire and normalized it by the number of SARS-CoV-2 epitopes (20) to be better able to compare to responses for other viruses.

**Fig. 6.**
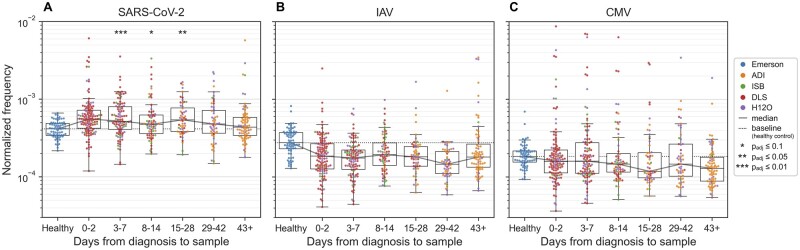
Analyzing TCR repertoires of COVID-19 patients with TCRconv. Dynamics of (**A**) SARS-CoV-2 (**B**) IAV and (**C**) CMV-specific T cells in terms of frequency normalized by the number of virus-related epitopes. There are 20 epitopes for SARS-CoV-2, 8 for IAV and 9 for CMV. Each data point corresponds to a repertoire and is colored by its dataset (Supplementary Table S4). Symbols “*” indicate statistically significant increase in frequency compared to healthy samples (see Supplementary Table S5)

Figure 6A shows that the frequency of SARS-CoV-2-specific T cells is highest during the first two days after diagnosis and starts to decrease later after the infection. In contrast, with influenza A virus (IAV), cytomegalovirus (CMV), Epstein-Barr virus (EBV) and hepatitis C virus (HCV) (Fig. 6 and Supplementary Fig. S12), the normalized frequency remains lower.

To assess if the COVID-19 patients have significantly higher frequency of virus-specific T cells than healthy control subjects, and if the frequencies are positively correlated with subjects’ age, a linear regression analysis was performed. This was done separately for each time interval using linear model $y=a+b_{\text{cc}}x_{\text{cc}}+b_{\text{age}}x_{\text{age}}$, where *y* is the observed frequency, *a* is offset, $b_{\text{cc}}$ is parameter for case-control covariate $x_{\text{cc}}$ which is zero for control samples and one for case samples of the considered time interval and $b_{\text{age}}$ is parameter for age covariate $x_{\text{age}}$. This analysis showed that the case-control difference is significant for time intervals 3–7, 8–14 and 15–28, as quantified by the Benjamini–Hochberg corrected one-tailed *t*-test (see Fig. 6A and Supplementary Table S5).

#### 3.5.3 Phenotypes of SARS-CoV-2-specific TCRs

**Fig. 7.**
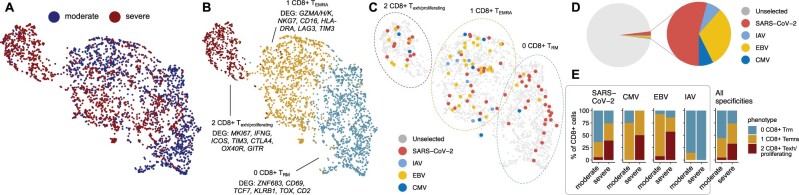
Characteristics of SARS-CoV-2-specific CD8+ T cells from bronchoalveolar lavage samples from patients with moderate (*n* = 3) or severe (*n* = 6) COVID-19 disease. UMAP presentations colored by (**A**) disease severity and (**B**) phenotypes. Panel (**C**) shows clustering with epitope-specific T cells marked, (**D**) proportions of epitope specific T cells and (**E**) phenotype distribution of virus specific T cells

To link TCR-specificity to T cell phenotype, we utilized scRNA+TCRαβ-seq of CD8+ T cells from bronchoalveolar lavage samples of nine COVID-19 patients with moderate or severe disease (Liao *et al.*, 2020). The scRNA-seq data were analyzed mainly with Python package scVI tools and R package Seurat (see Supplementary Section S4 for details). The T cell clustering colored by disease severity, phenotype and predicted epitope specificity is shown in Figures 7A–C As expected, SARS-CoV-2-specific T cells were highly abundant in these samples (Fig. 7D). Interestingly, consistent with slightly elevated frequency of EBV-specific T cells in COVID-19 repertoires (Supplementary Fig. S12C), T cells specific to EBV epitopes were also abundant in bronchoalveolar lavage samples. Moreover, in patients with moderate disease (*n* = 3) the SARS-CoV-2-specific T cells most often had tissue-resident memory phenotype (overexpression of ZNF683, CD69, TCF7) (Fig. 7A–C). In patients with severe disease (*n* = 6), we found SARS-CoV-2-specific T cells to have possibly overtly proliferating (MKI67) and exhausted (HAVCR2/TIM3, CTLA4) phenotype, with high expression of co-stimulatory signals (ICOS, TNFRSF4/OX40R, GITR) and IFNG (Fig. 7A–C and E). These findings refine previous findings of Moss (2022) by suggesting that patients with a moderate disease course form T cells capable of eliminating SARS-CoV-2 with minimal tissue damage while T cell overactivation in patients with a severe disease leads to an inappropriate tissue damage. The patient from which the T cells originate, and the frequency of epitope-specific TCRs per patient are shown in Supplementary Figure S13.

### 3.6 Experimental validation of TCRconv predictions

**Fig. 8.**
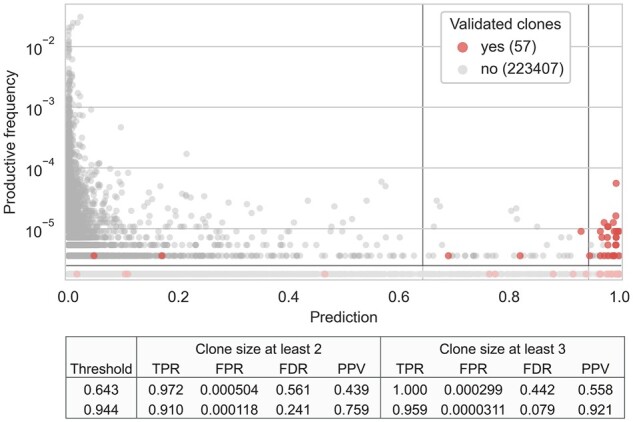
Predicted and experimentally validated specificity of TCRs for SARS-CoV-2 epitope Spike_YLQPRTFLL_. Each TCR clone in the repertoire sample ADIRP0000466_20200518 is represented as a circle that is colored red if it has been validated in the MIRA experiment eJL158 and grey if not. Each circle is positioned by it’s productive frequency (*y*-axis) and TCRconv prediction score (*x*-axis). The two vertical black lines show prediction thresholds 0.643 and 0.944 that correspond to false positive rates of 0.001 and 0.0001 obtained from the 10-fold cross-validation with VDJdbβ-large dataset. The TCRs with clone size one are shaded. The table below shows the true positive rate (TPR), false positive rate (FPR), false discovery rate (FDR) and positive predictive value (PPV) for the two thresholds and for clones of size at least two or at least three (A color version of this figure appears in the online version of this article)

To estimate how well TCRconv performs compared to experimental measurements, we again utilized ImmuneCODE samples (Nolan *et al.*, 2020) for which both a TCRβ repertoire sequencing and a MIRA experiment were reported. We opted to focus on the Spike_YLQPRTFLL_ epitope that was the only SARS-CoV-2 epitope in our VDJdbβ-large dataset, and used the TCRconv predictor trained on that dataset to make predictions for the TCR repertoires. One of the peptide groups used in the MIRA experiments consisted of peptides YLQPRTFL, YLQPRTFLL and YYVGYLQPRTF. Here we assume that a TCR that is found to recognize one of these peptides in the MIRA experiment is likely to recognize Spike_YLQPRTFLL_. We only focused on the TCR clones from the repertoires with size two or larger, so that there would be a reasonable chance of capturing the exact same TCR both in the repertoire and the MIRA experiment (see Fig. 8). To be able to evaluate TCRconv’s prediction accuracies, we analyzed repertoire and MIRA sample pairs, where at least five TCR clones from the repertoire (with size two or larger) were validated in the corresponding MIRA experiment.

Figure 8 shows the TCRconv predictions for each TCR clone in a repertoire together with the experimentally validated Spike_YLQPRTFLL_-specific TCRs. We computed four metrics for evaluating this performance: true positive rate (TPR), false positive rate (FPR), false discovery rate (FDR) and positive predictive value (PPV). These results show that TCRconv is able to identify a large portion of the Spike_YLQPRTFLL_-specific TCR clones that are validated in the matched MIRA experiment. Furthermore, the proportion of false positive predictions is small, especially when only larger clone sizes are considered. Supplementary Figure S14 results for another pair of matched repertoire and MIRA experiments.

## 4 Discussion and conclusions

Here, we have presented TCRconv, a novel deep learning method that combines transformer embeddings for TCR sequences and a CNN predictor. The protBERT model transfers useful information to the CDR3 embedding from the TCR regions surrounding it, making a compact and rich presentation of the TCR. Unlike previous methods, it has been formulated as a multilabel predictor that can make predictions simultaneously for multiple epitopes and account for cross-reacting TCRs. We have demonstrated that TCRconv has state-of-the-art accuracy in terms of AUROC and AP scores. We have also demonstrated how TCRconv can be applied for unsupervised analysis of TCR-repertoires as well as to link T cell phenotypes and epitope-specificity with single-cell RNA+TCR-seq data.

Machine learning methods in general benefit from having ample data for training the models. As the amount of epitope-specific data increases the accuracy of existing methods such as TCRconv will further improve. Similarly, having sufficiently long sequencing reads that allows the recovery of the complete V(D)J sequences can clearly benefit the predictors, as we showed with the varying context sizes. As also the number of unique epitopes increases, it can become more productive to also incorporate the epitope sequence into TCR-epitope prediction models and predict TCRs’ specificity to previously unseen epitopes.

## Supplementary Material

btac788_Supplementary_Data

## Data Availability

Implementation for TCRconv and the epitope-specific TCR datasets are available at https://github.com/emmijokinen/tcrconv. The VDJdbβ-large, VDJdbβ-small, and VDJdbαβ-large datasets were obtained from the VDJdb database by Bagaev et al. (2020) (https://vdjdb.cdr3.net). Additional SARS-COV-2 specific TCRs and COVID-19 patient repertoires are available in the immuneACCESS database at https://clients.adaptivebiotech.com/pub/covid-2020. Control repertoires from Emerson et al. (2017) are available in the immuneACCESS database at https://doi.org/10.21417/B7001Z. Implementation for the scRNA+TCRab-seq data analysis is available at https://github.com/janihuuh/tcrconv_manu. Count matrices, TCRαβ-seq results, and metadata from Liao et al. (2020) are available at GEO GSE145926.
