# Supplementary material for: TCRconv: predicting recognition between T cell receptors and epitopes using contextualized motifs
Source: Bioinformatics. 2022 Dec 7;39(1):btac788. doi: 10.1093/bioinformatics/btac788 (PMC9825763; doi:10.1093/bioinformatics/btac788)
Supplement: btac788_Supplementary_Data [file btac788_supplementary_data.pdf]

Supplementary material

TCRconv: Predicting recognition between T cell receptors and epitopes using contextualized motifs

S1. VDJdb confidence scores

All TCR-epitope pairs in VDJdb have been given confidence scores from 0-3 as follows:

0: low confidence or no information (a critical aspect of sequencing or specificity validation is missing)
1: moderate confidence (no verification or poor TCR sequence confidence)
2: high confidence (has some specificity verification, good TCR sequence confidence)
3: very high confidence (has extensive verification or structural data)

See more detailed description at https://github.com/antigenomics/vdjdb-db

S2. Other embedding techniques for TCRs

We also attempted to make the ProtBERT model more specialized to TCR sequences by fine-tuning it on 5 million TCRβ sequences from VDJdb (Bagaev, *et al.*, 2020), and studies of Emerson *et al.* (2017) and Dash *et al.* (2017) for eight epochs but this did not improve the prediction accuracies (mean AUROC 0.848 and AP 0.575 on VDJdbβ-small dataset). We also tested two ELMo (Embeddings from Language Models) architectures, classical ELMo (Peters *et al.*, 2018) and masked ELMo (Senay and Salin, 2020), and trained them on a smaller dataset of 3 million TCRβ-sequences from the same sources as those used in the BERT fine-tuning. The main difference between these two models is that instead of unidirectional LSTMs, the masked ELMo uses a bidirectional two-layered LSTM and when trained in the token prediction task, the predicted token (amino acid) is masked to avoid leakage of information. We found that both ELMo models produced reasonable accuracies in the prediction task, and with masked ELMo we achieved almost as good accuracy as with the BERT embeddings (mean AUROC and AP 0.838 and 0.539 for ELMo and 0.847 and 0.571 for masked ELMo, on VDJdbβ-small dataset).

S3. Saliency maps

Gradient-based saliency maps can describe how much each position in a sequence influences the predicted epitope-specificity. We computed saliency maps for a TCRconv model with the protBERT model for computing embeddings for the CDR3 sequences using the full context (i.e., an embedding is first computed for the complete TCR determined by the CDR3, and V- and J-genes, and then the part corresponding to the CDR3 is extracted), trained with the VDJdbαβ-large dataset. The saliency values were computed as the average over all absolute saliency values for all features at each position. To determine the importance of each residue individually, we compute the gradients of the true epitope binding w.r.t. the outputs of the non-contextualized layer (the input layer embedding's output) of protBERT. The values were scaled between 0 and 1 for each TCR separately. We chose to extract contextualized embeddings with protBERT without further fine-tuning its parameters (we did not find improvements when doing so, see Supplementray Section S2, and we wanted to avoid overfitting). Therefore, the gradients with respect to individual, uncontextualized residues need to propagate through the 30 untuned transformer layers in protBERT. Due to the complexity of the protBERT model, the high dimensionality of the embeddings, and the multiple convolutional layers in our predictor (four parallel convolutional layers and another consecutive convolutional layer, each with several filters), it is expected that identification of clear motifs can be challenging. This is illustrated by Supplementary Figures S15-S16 that show examples of these saliency maps for TCRs recognizing seven different epitopes, each from different epitope species. However, some more general observations can be made; On average the position-wise saliency values for the positions in CDR3 are higher than those outside the CDR3, and with the paired TCRαβ, the average position-wise saliency was in general higher for the chain that had better predictive performance when used individually (see Supplementary Table S7 and Fig. 5). A few examples of saliency maps for paired TCRαβ sequences are shown in Supplementary Fig. S17.

S4. Phenotypes of SARS-CoV-2 specific T cells in moderate and severe COVID-19

Count matrices, TCRαβ-seq results, and metadata from Liao *et al.* (2020) were downloaded from GEO GSE145926. The data was analyzed mainly with Python package scVI tools (Gayoso *et al.*, 2022) (v 0.14.5) and R package Seurat (Hao *et al.*, 2021) (v 4.0.4). Cells with > 10% mitochondrial gene counts, < 1000 UMI counts, < 200 or > 6000 detected genes, and cells with no detected TCR were filtered out. The highly variable genes were identified with “highly_variable_genes” function in scVI tools with default parameters, which were then used to learn latent embeddings with “model.SCVI” function in scVI tools with default parameters. The CD8+ T cells were then identified with SingleR (Aran *et al.*, 2019) (v 1.6.1), and the process was repeated with scVI tools. The obtained embeddings were then used for finding clusters with “FindNeighbors” and “FindClusters” functions and further visualized with UMAP dimensionality reduction with “RunUMAP” function using default parameters in Seurat. The optimal clustering threshold was chosen as 0.2 based on visual inspection of the clustering results in the UMAP reduced space. The markers used to define the clusters were found with Student's t-test using the “FindMarkers” function in Seurat with logfc.threshold = 0.25 from expression data that was scaled with “ScaleData” function with scaling factor of 10000. Patients C141, C142, and C144 have moderate COVID-19. Patients reported by Liao *et al.* (2020) to have severe (C143 and C145) or critical disease (C146, C148, C149, and C152) were considered to have severe COVID-19 in these analyses.

S5. Box plots

All boxplots presented in the paper have the same formatting, that uses the default settings used by Seaborn[1] and Matplotlib[2] Python packages for the extents of the box, center line, and whiskers. That is, the box extends from the first quartile (Q1) to the third quartile (Q3) of the data and the median is marked with a line. The whiskers extend from the box by 1.5 times the inter quartile range. The whiskers are thus placed at Q1 - 1.5 × (Q3 - Q1) and Q3 + 1.5 × (Q3 - Q1). Addititionally, all datapoints are shown, see the Figure legends for their descriptions.

1. https://seaborn.pydata.org/generated/seaborn.boxplot.html
2. https://matplotlib.org/stable/api/_as_gen/matplotlib.pyplot.boxplot.html

References

Aran, D. *et al.* (2019). Reference-based analysis of lung single-cell sequencing reveals a transitional profibrotic macrophage. *Nature immunology*, **20**(2), 163–172.

Bagaev, D. V. *et al*. (2020). VDJdb in 2019: database extension, new analysis infrastructure and a T-cell receptor motif compendium. *Nucleic Acids Research*, **48**(D1), D1057–D1062.

Dash, P. *et a*l. (2017). Quantifiable predictive features define epitope-specific T cell receptor repertoires. *Nature*, **547**(7661), 89–93.

Emerson, R. O. *et al.* (2017). Immunosequencing identifies signatures of cytomegalovirus exposure history and HLA-mediated effects on the T cell repertoire. *Nature Genetics*, **49**(5), 659–665.

Gayoso, A. *et al.* (2022). A python library for probabilistic analysis of single-cell omics data. *Nature Biotechnology*, **40**(2), 163–166.

Hao, Y. *et al.* (2021). Integrated analysis of multimodal single-cell data. *Cell*, **184**(13), 3573–3587.

Liao, M. *et al.* (2020). Single-cell landscape of bronchoalveolar immune cells in patients with COVID-19. *Nature medicine*, **26**(6), 842–844.

Peters, M. E. et al. (2018). Deep contextualized word representations. *arXiv*.

Senay, G. and Salin, E. (2020). Masked ELMo: An evolution of ELMo towards fully contextual RNN language models. *arXiv*.

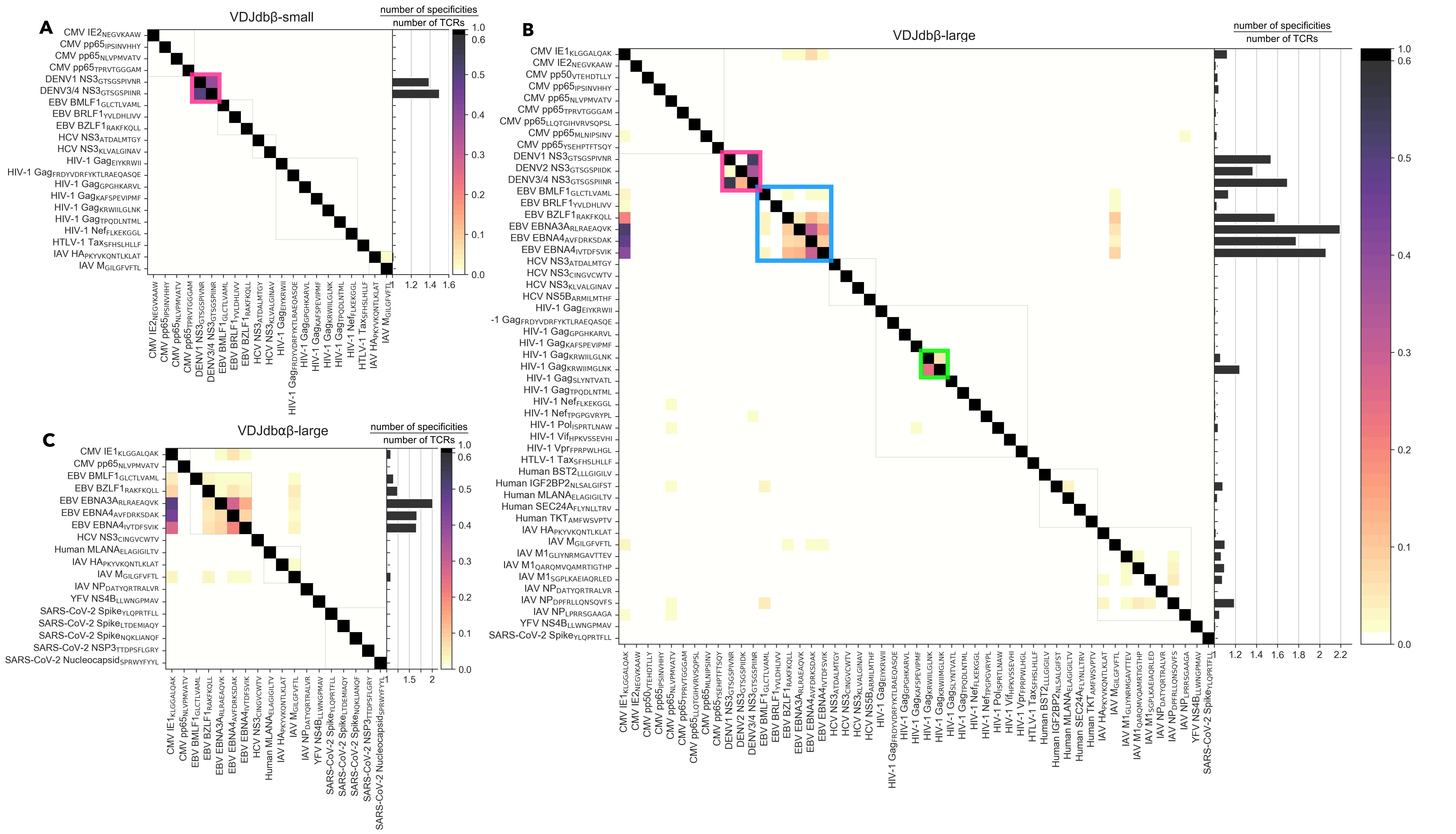

Supplementary Fig. S1. TCR cross-reactivity in datasets a) VDJdbβ-small, b) VDJdbβ-large, and c) VDJdbaβ-large. Each row of a heat map represents TCRs specific to the corresponding epitope and their fraction recognizing any of the epitopes present in the dataset. The bar plots on the right side of each heatmap show the average number of epitope specificities per TCR recognizing the epitope on the corresponding row. For example, TCRs specific to EBV epitope EBNA3A$_{RLRAEAQVK}$ recognize on average 2.2 different epitopes on (b) dataset VDJdbβ-large and 2.0 on (c) dataset VDJdbαβ-large. TCRs recognizing certain epitopes have notable cross-reactivity. To highlight them we have marked DENV epitopes with pink, EBV epitopes with blue, and two HIV-1 epitopes (HIV-1$_{KRWIILGLNK}$ and HIV-1$_{KRWIIMGLNK}$) with green.

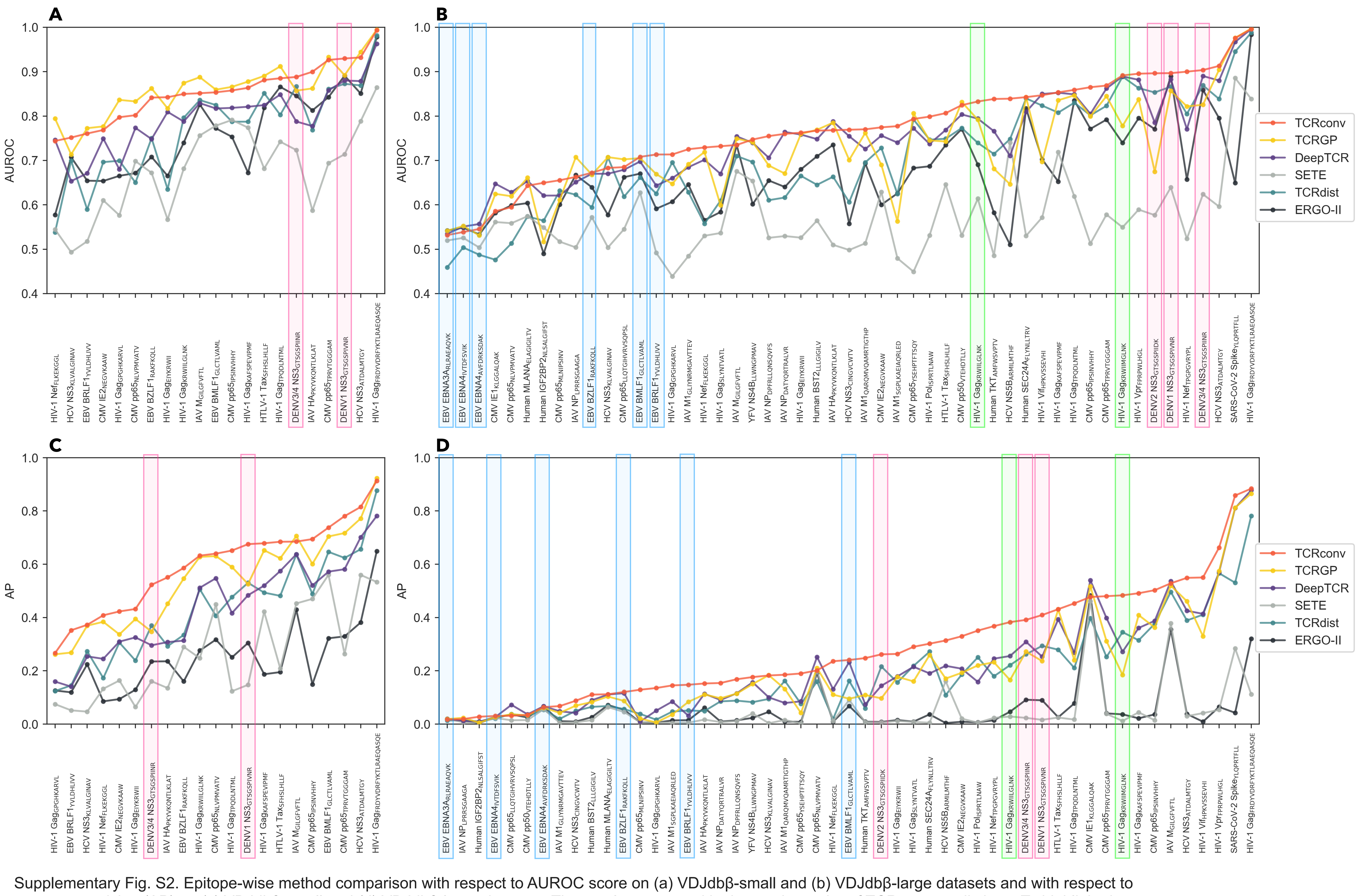

Supplementary Fig. S2. Epitope-wise method comparison with respect to AUROC score on (a) VDJdbβ-small and (b) VDJdbβ-large datasets and with respect to average precision (AP) on (c) VDJdbβ-small and (d) VDJdbβ-large datasets. The results are sorted by increasing order of TCRconv predictions. To highlight the accuracies for epitopes with notably cross-reactive TCRs, we have highlighted such epitopes similarly to Supplementary Fig. S1: DENV epitopes with pink, EBV epitopes with blue, and two HIV-1 epitopes (HIV-1$_{KRWIILGLNK}$ and HIV-1$_{KRWIIMGLNK}$) with green.

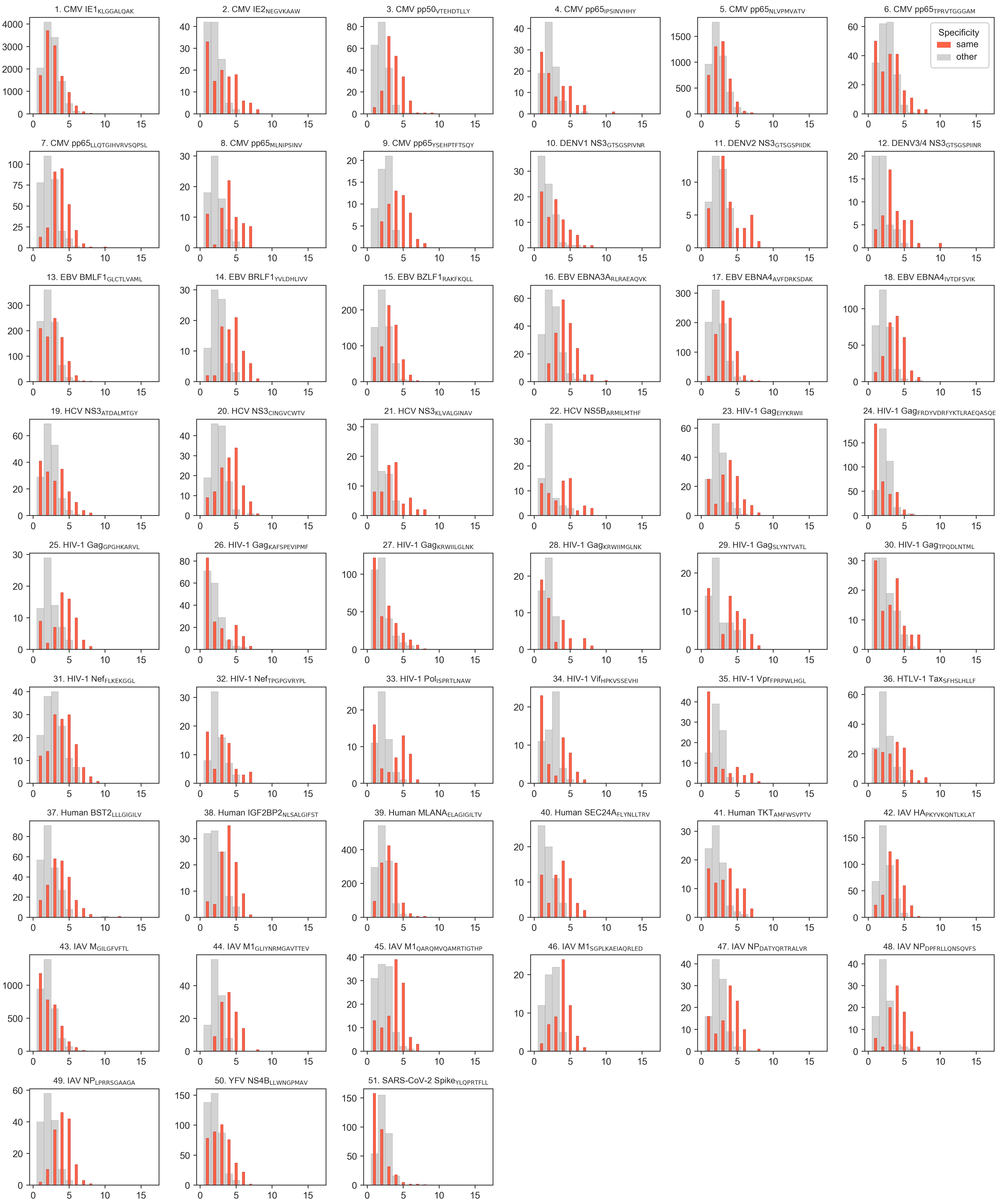

Supplementary Fig. S3. CDR3 edit distances on VDJdbβ-large from TCRs with chosen specificity to nearest TCR with same specificity (red) or other specificity (grey).

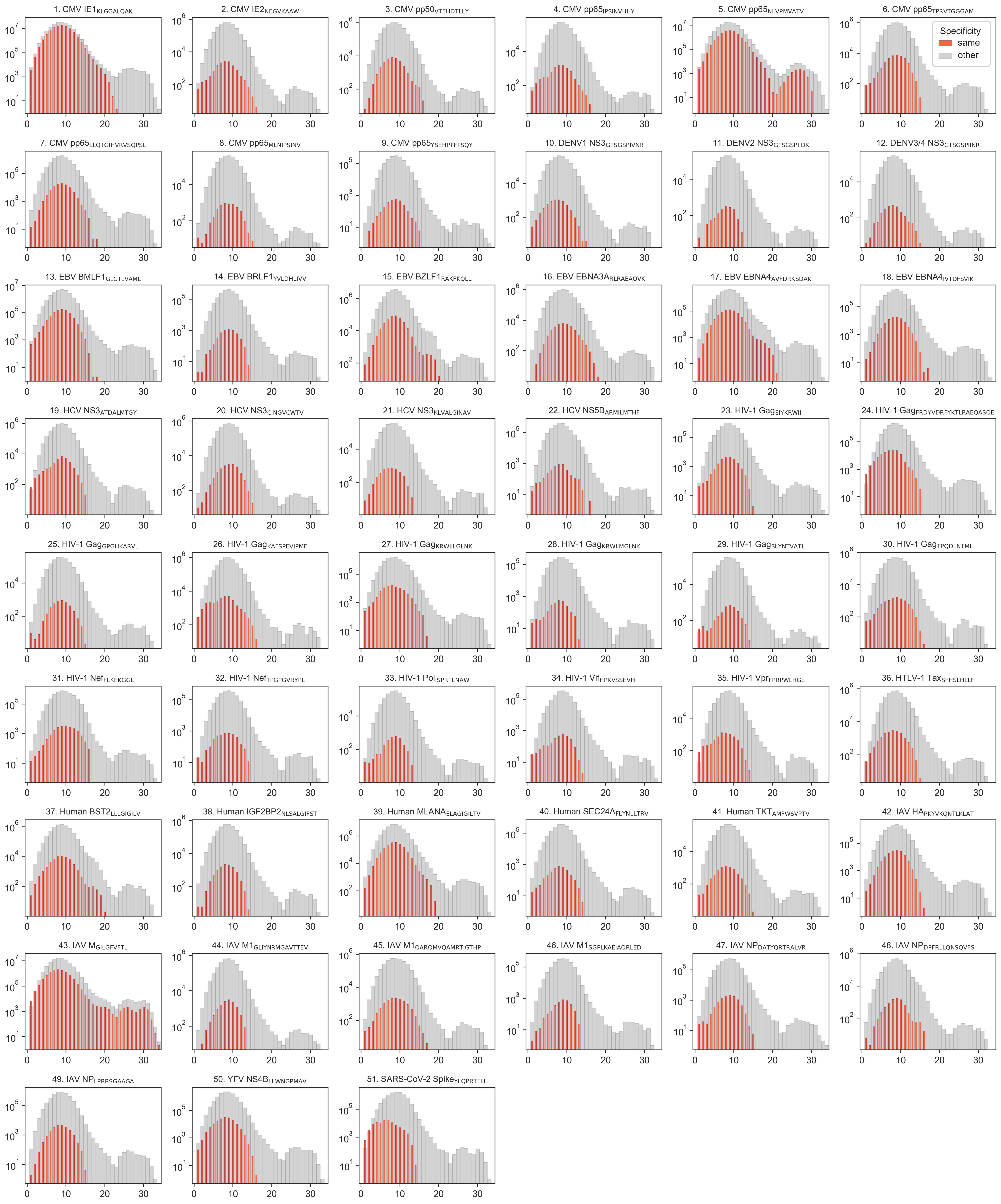

Supplementary Fig. S4. CDR3 edit distances on VDJdbβ-large from TCRs with chosen specificity to all TCRs with same specificity (red) or to all TCRs with other specificity (grey). Y-axis has log-scale.

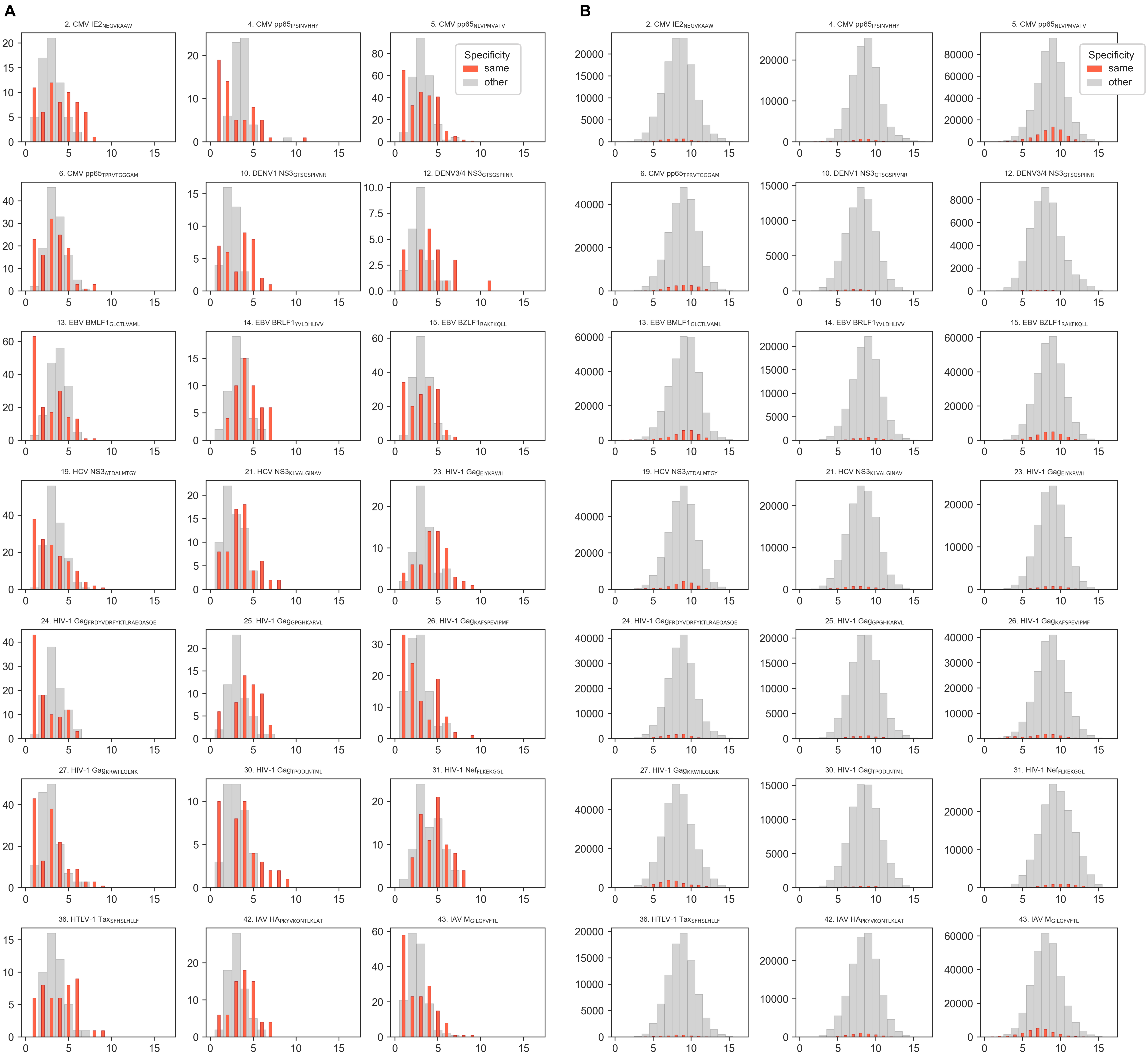

Supplementary Fig. S5. CDR3 edit distances on VDJdbβ-small. (A) Edit distance from TCRs with chosen specificity to nearest TCR with same specificity (red) or other specificity (grey). (B) CDR3 edit distance from TCRs with chosen specificity to all TCRs with same specificity (red) or to all TCRs with other specificity (grey)

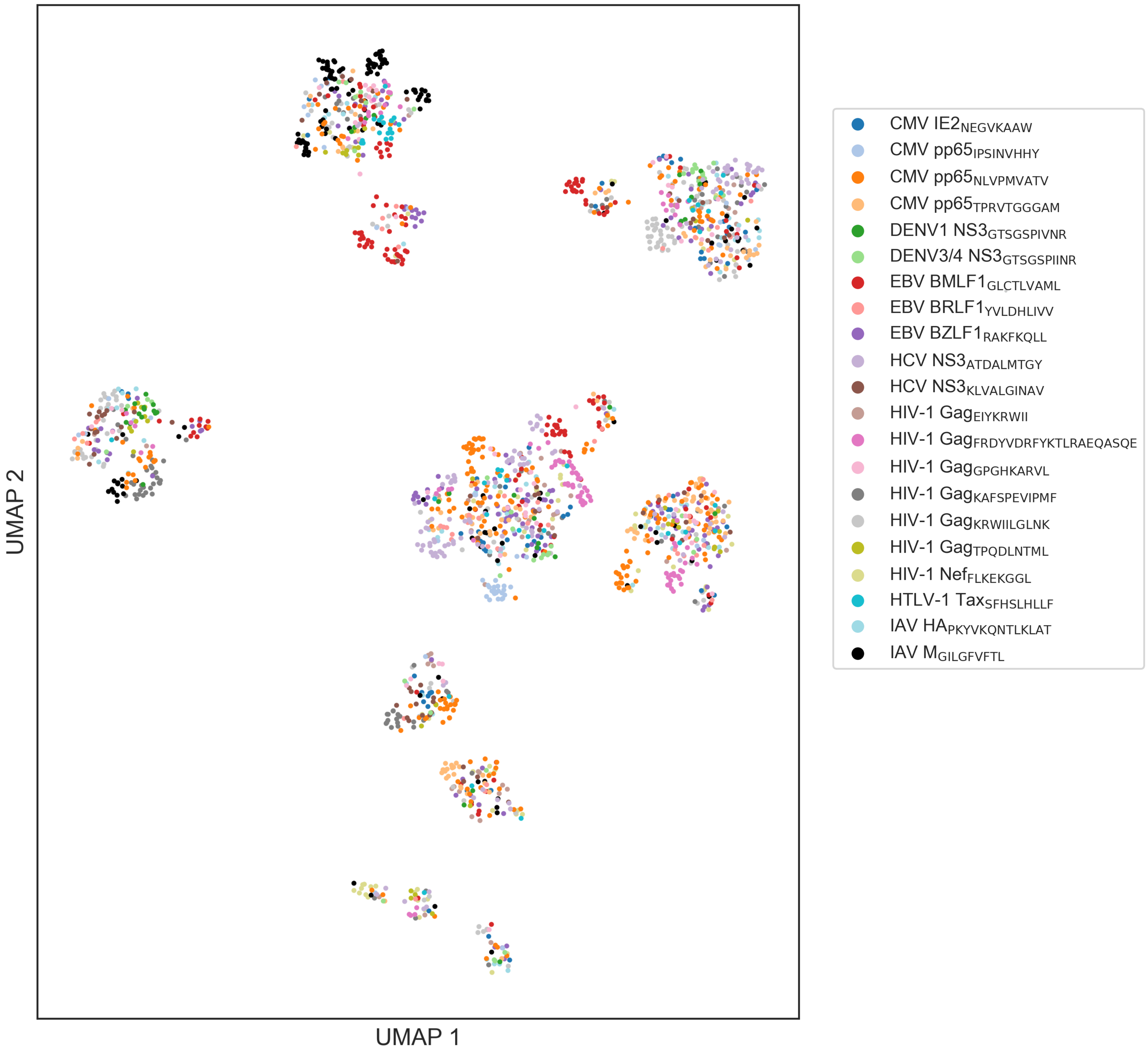

Supplementary Fig. S6. UMAP clustering of TCRs in VDJdbβ-small. Each dot correpsonds to one TCR and is colored by its epitope specificity. TCRs specific to multiple epitopes are colored by only one of its specificities.

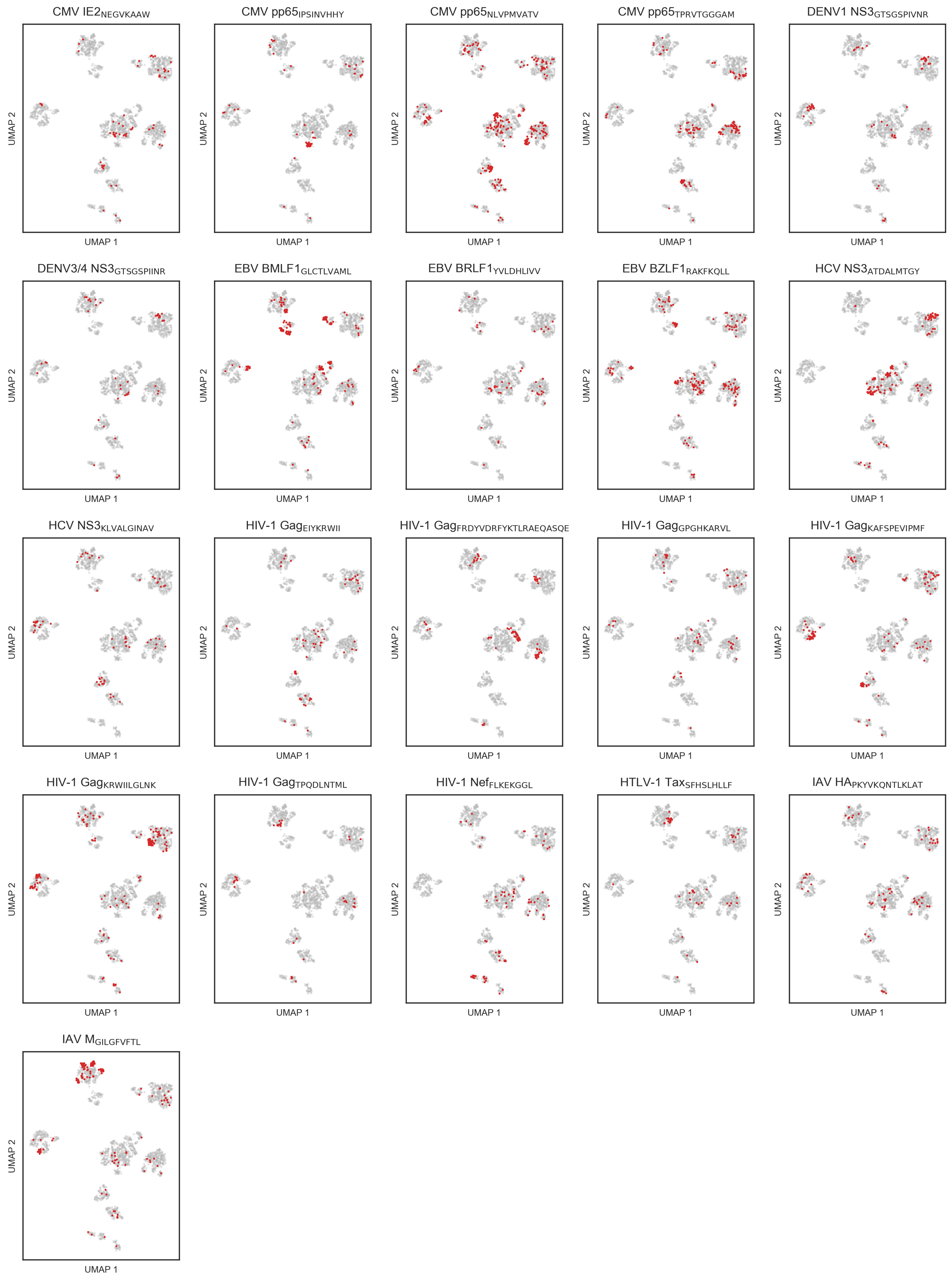

Supplementary Fig. S7. UMAP clustering of TCRs in VDJdbβ-small. Each dot correpsonds to one TCR and is colored with red if it recognizes the epitope in the title and otherwise with grey.

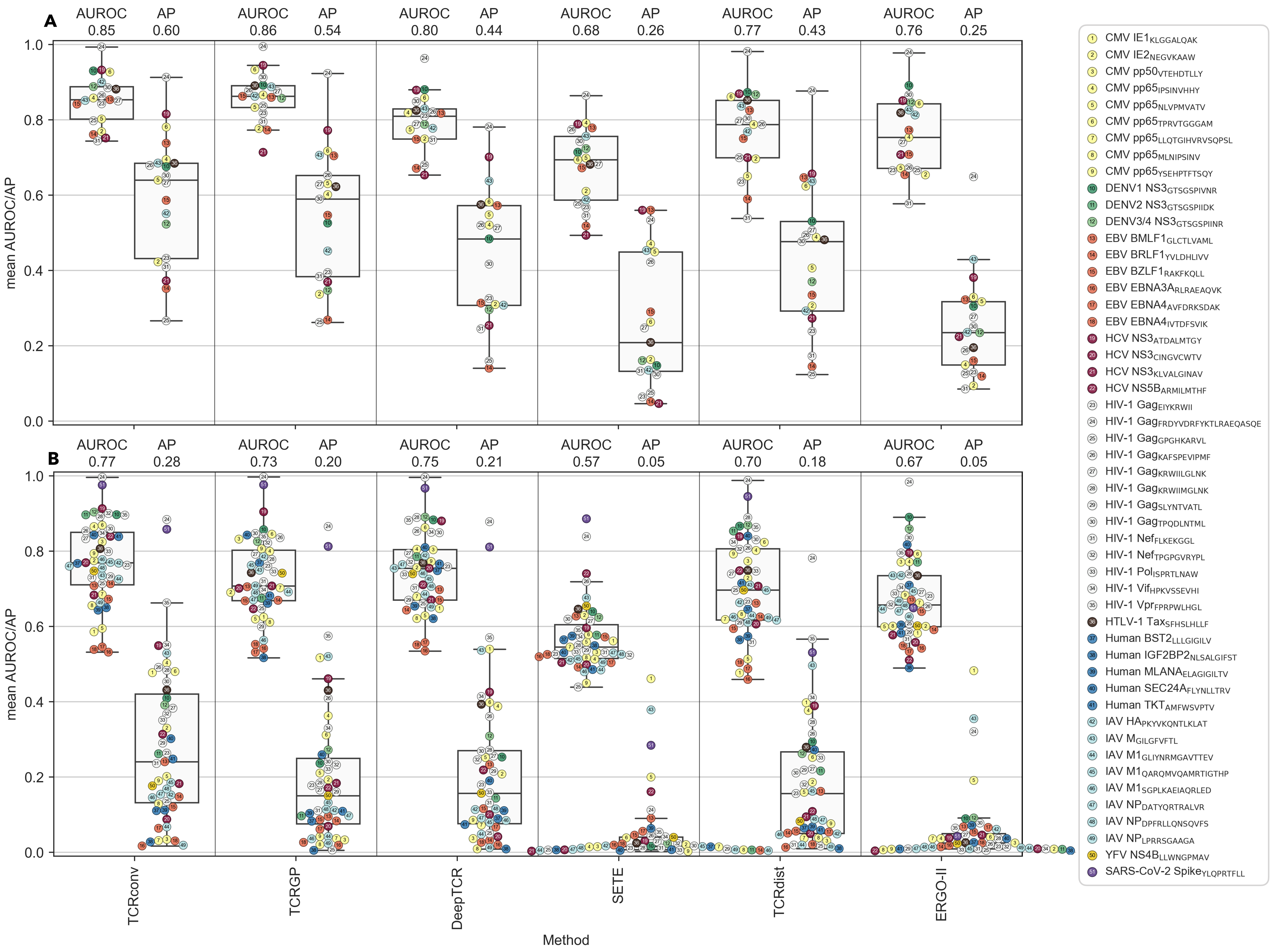

Supplementary Fig. S8. Method comparisons. Mean AUROC and AP scores on (a) VDJdbβ-small and (b) VDJdbβ-large dataset.

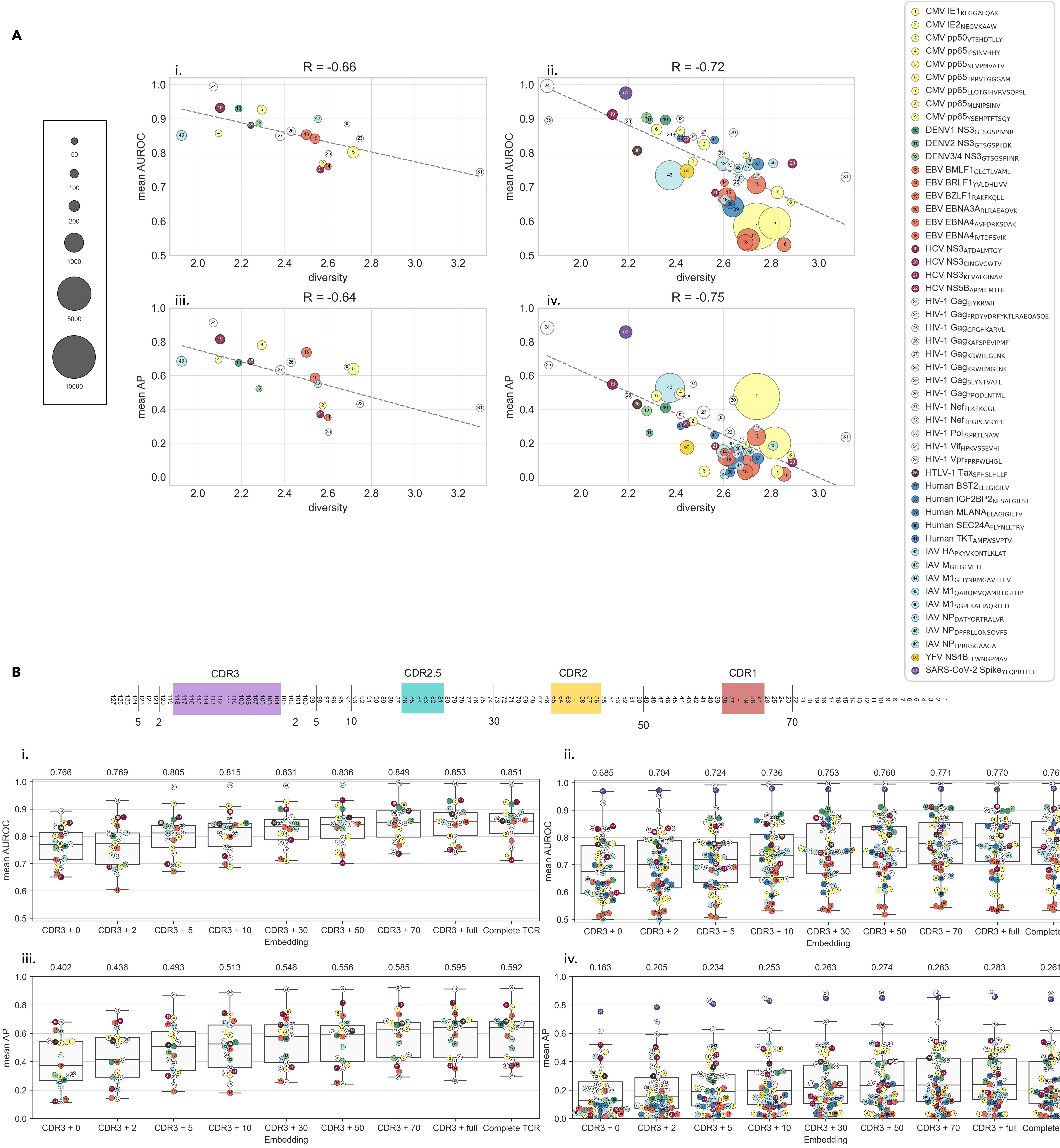

Supplementary Fig. S9. TCRconv evaluation. All AUROC and AP scores are obtained over stratified 10-fold cross-validation.
(A) Pearson's correlation between the diversity of epitope specific TCRs and the AUROC and AP scores. Panels (i) and (ii) show the mean AUROC scores for datasets VDJdbβ-small and VDJdbβ-large, respectively, and (iii) and (iv) mean AP scores for both datasets.
(B) Increasing embedding context size increases the predictive AUROC and AP scores. The schematics on the top show the approximate sections included in different context sizes. Complete TCR refers to using the complete TCR with the predictor, without extracting only the CDR3 part. Panels (i) and (ii) show the mean AUROC scores for datasets VDJdbβ-small and VDJdbβ-large, respectively, and (iii) and (iv) mean AP scores for both datasets.

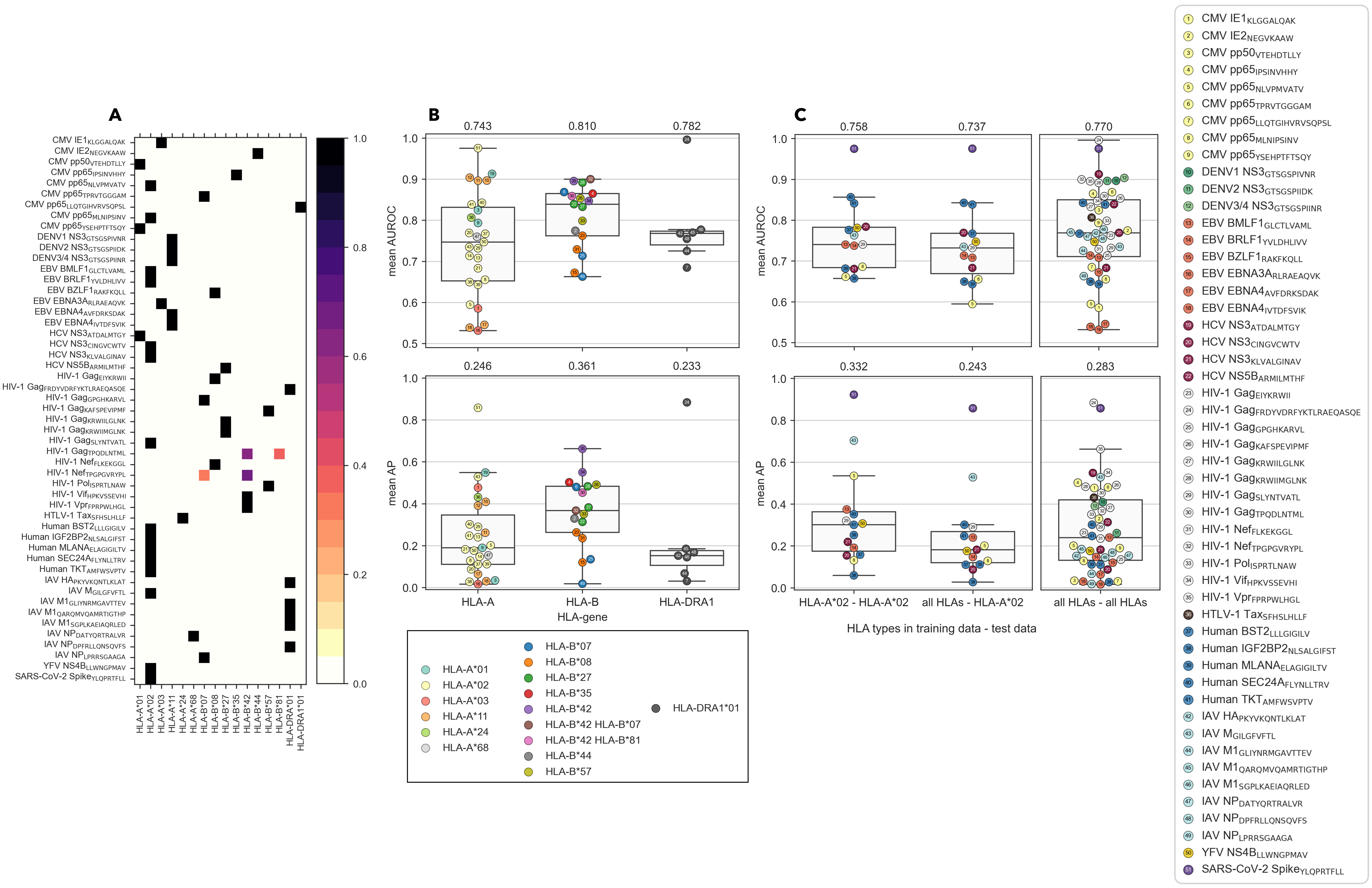

Supplementary Fig. S10. HLA-types of the MHCs restricting the epitopes do not alone explain variance in results. All AUROC and AP scores are obtained over stratified 10-fold cross-validation.

(A) HLA-types of the MHCs restricting the epitopes in dataset VDJdbβ-large.

(B) TCRconv predictions for VDJdbβ-large dataset have some variation in terms of AUROC and AP scores when the predictions are divided into three groups (HLA-A, HLA-B, and HLA-DRA1) based on the HLA-gene.

(C) AUROC and AP scores for HLA-A*02 restricted epitopes are similar whether the TCRconv is trained only on TCRs specific to HLA-A*02 restricted epitopes or to TCRs specific to all epitopes in VDJdbβ-large dataset. For reference TCRconv model trained and tested with TCRs specific to all epitopes, corresponding to results shown in Fig. 1a ii, is shown on right.

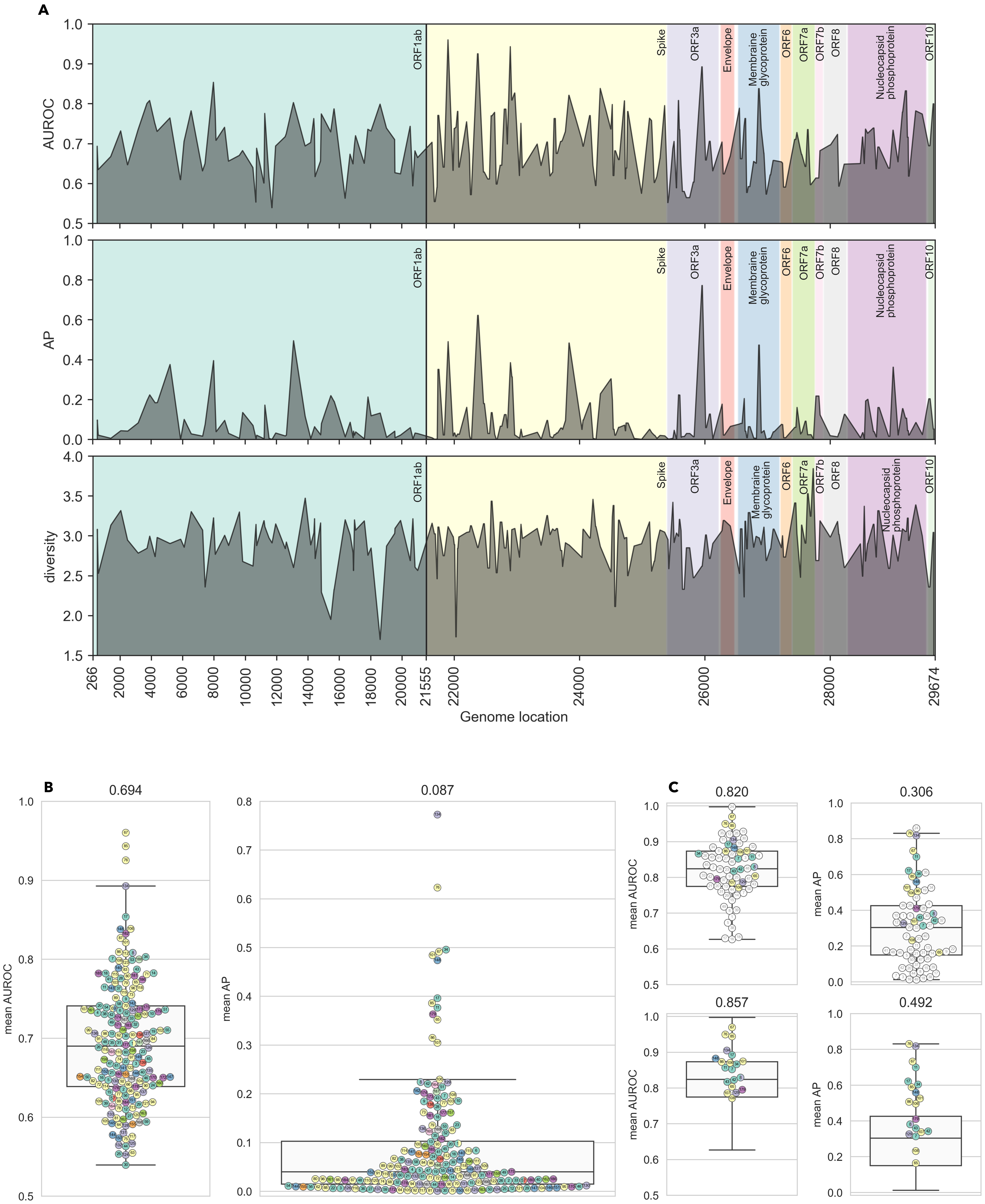

Supplementary Fig. S11. TCRconv prediction performance for SARS-CoV-2 epitopes.

(A) TCRconv performance in terms of AUROC and AP scores when trained with 139099 TCRs specific to 188 peptide groups from SARS-CoV-2. Mean scores are shown above both boxplots. Each circle represents the score for one peptide group, colored by the genomic region and numbered according to Supplementary Table S3.

(B) TCRconv performance when trained with TCRs specific to 20 best performing peptides groups from SARS-CoV-2 combined with VDJdbβ-large dataset; above results for all 70 peptide (groups) and below for only the 20 SARS-CoV-2 peptides. For SARS-CoV-2 peptides coloring and numbering are the same as in panel (a), other epitopes are white, and the numbering corresponds to Supplementary Table S1.

(C) AUROC and AP scores from the model from (a) by the peptides' genome location and the diversity of the TCRs specific to each peptide group by the peptides' genome location.

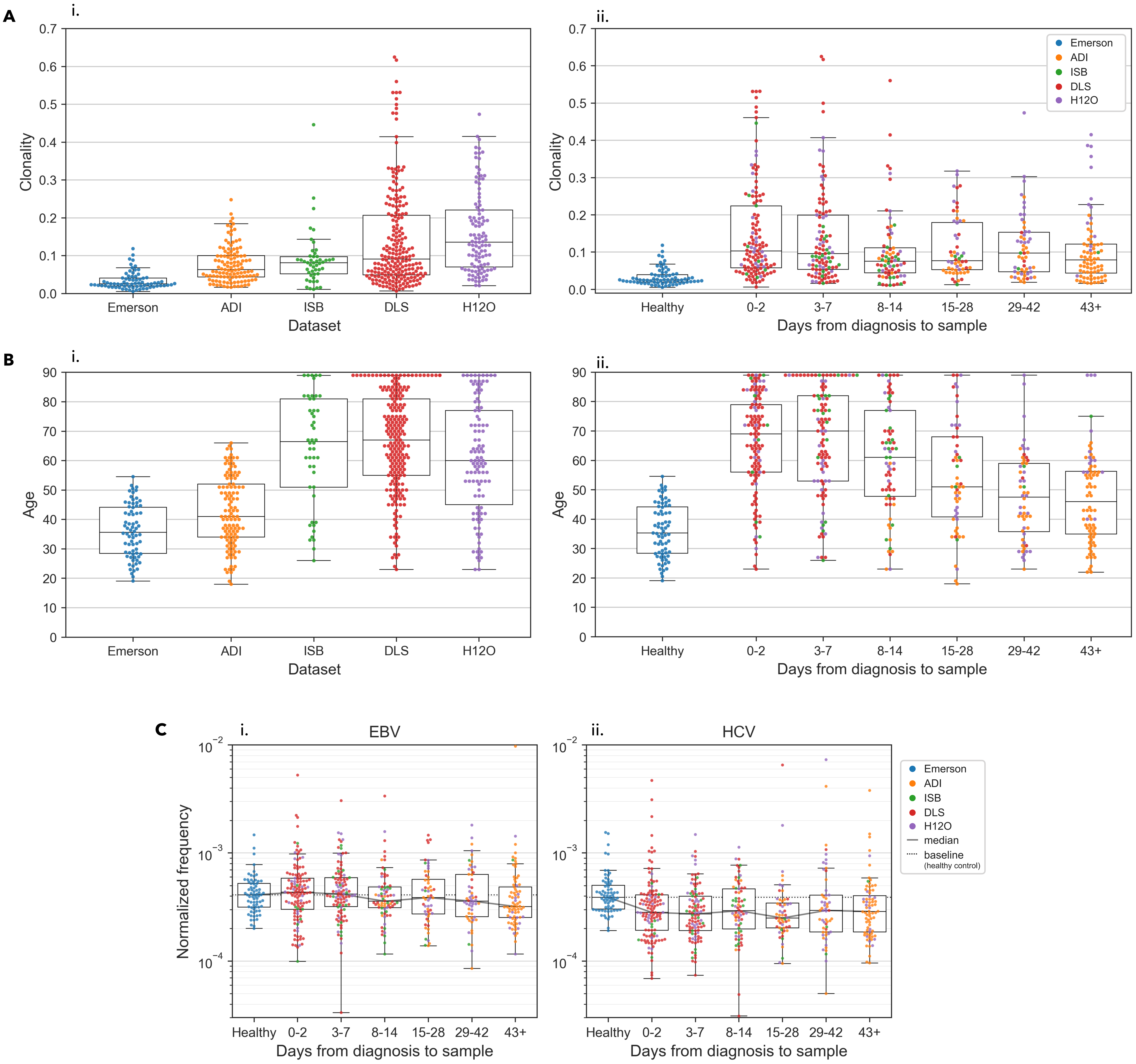

Supplementary Fig. S12. Analysis with COVID-19 patient repertoires.
(A) Shannon clonality (i) for each dataset and (ii) by Days from diagnosis to sample.
(B) Subject age (i) by dataset and (ii) by Days from diagnosis to sample.
(C) Normalized frequency grouped by number of days from diagnosis to sample for (i) six EBV specific epitopes and (ii) for four HCV specific epitopes.

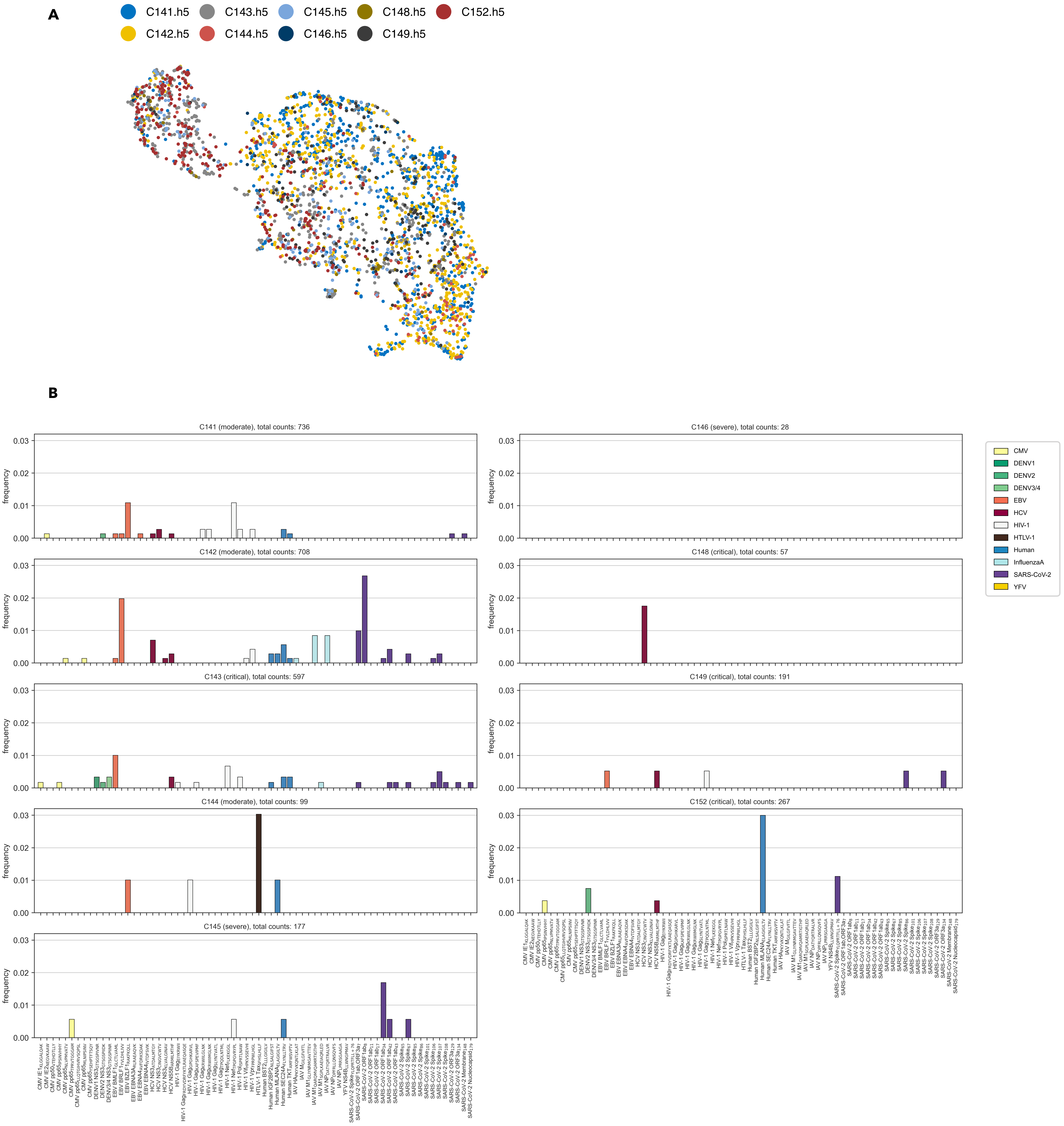

Supplementary Fig. S13. T cell phenotypes and specificity in COVID-19.
(A) Characteristics of scRNA+TCR UMAP representation of CD8+ T-cells based on their phenotypes, colored by patient. Patients C141, C142, and C144 have moderate COVID-19, while patients C143, C145, C146, C148, C149, and C152 have severe disease.
(B) Frequencies of T-cells predicted to be specific to the tested epitopes separately for each patient. Only T-cells with both TCR- and RNA-seq available are shown.

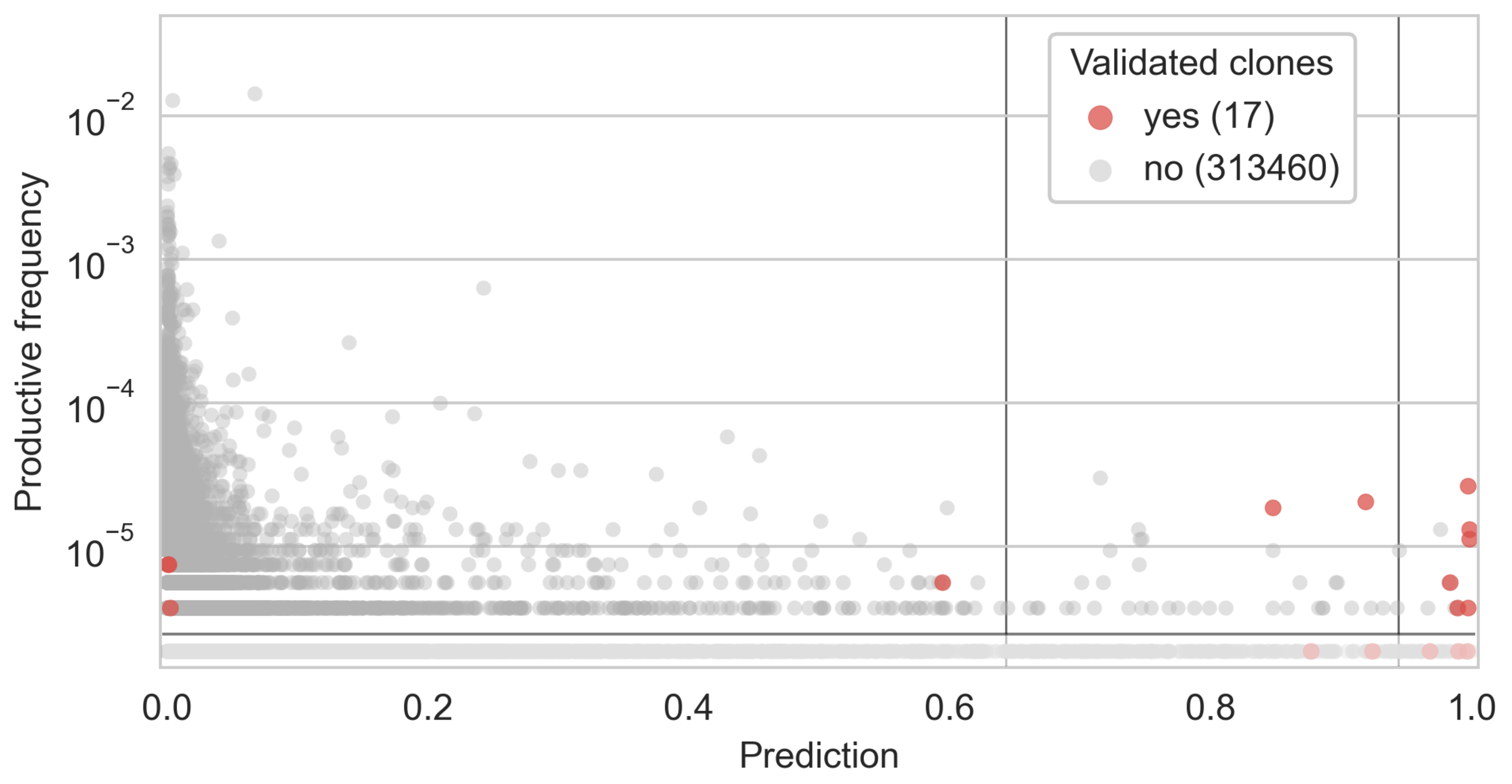

Threshold	Clone size at least 2				Clone size at least 3			
	TPR	FPR	FDR	PPV	TPR	FPR	FDR	PPV
0.643	0.809	0.000488	0.707	0.293	0.823	0.000391	0.608	0.392
0.944	0.500	0.0000770	0.382	0.618	0.484	0.0000743	0.333	0.667

Supplementary Fig. S14. Predicted and experimentally validated specificity of TCRs for SARS-CoV-2 epitope $\mathrm{Spike}_{\mathrm{YLQPRTFLL}}$.
Each TCR clone in the repertoire sample ADIRP0000273_20200527 is represented as a circle that is colored red if it has been validated in the MIRA experiment eQD123 and grey if not. Each circle is positioned by it's productive frequency (y-axis) and TCRconv prediction score (x-axis). The two vertical black lines show prediction thresholds 0.643 and 0.944 that correspond to false positive rates of 0.001 and 0.0001 obtained from the 10-fold cross-validation with VDJdbβ-dataset. The TCRs with clone size one are shaded. The table below shows the true positive rate (TPR), false positive rate (FPR), false discovery rate (FDR), and positive predictive value (PPV) for the two thresholds and for clones of size at least two or at least three.

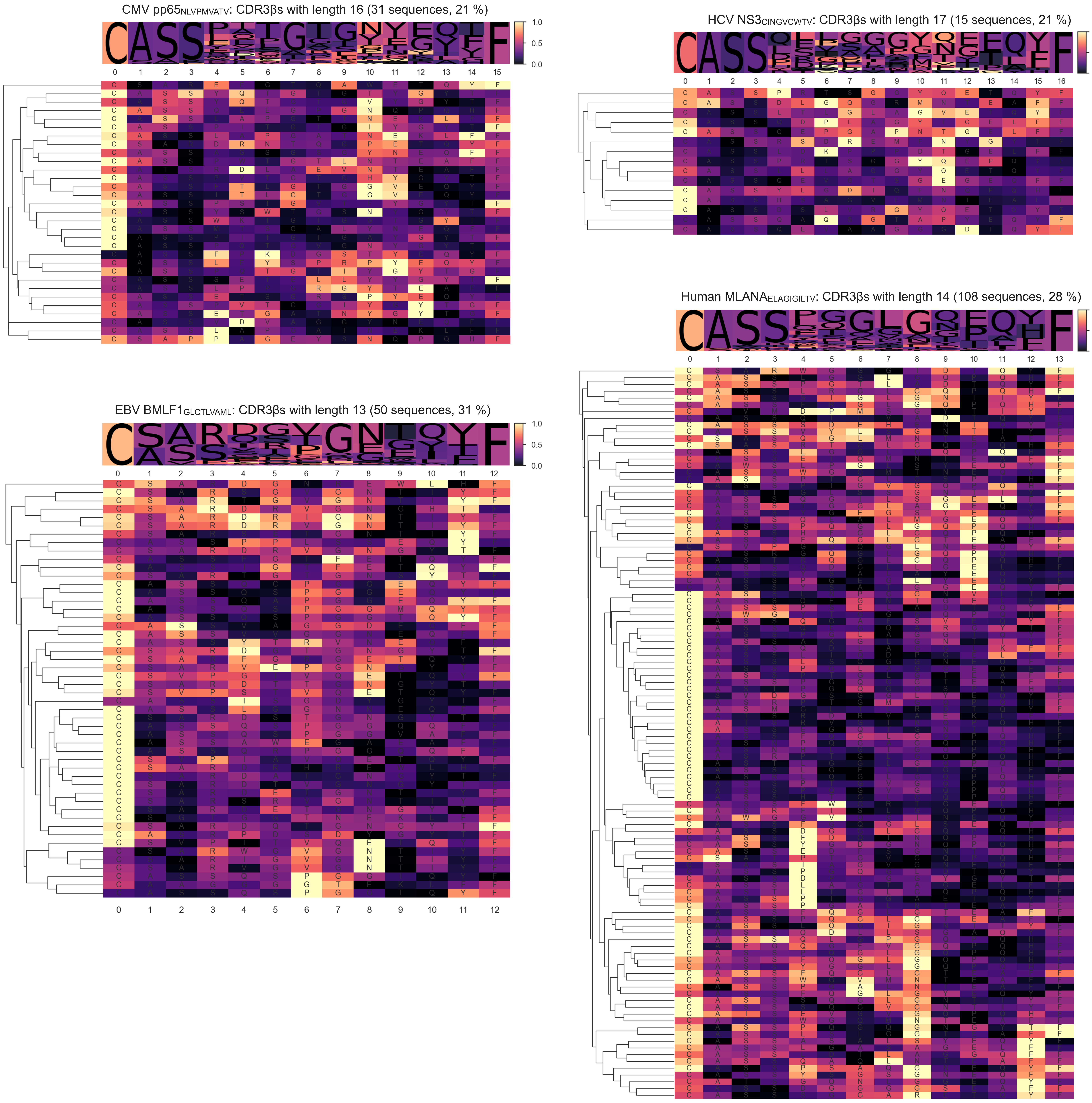

Supplementary Fig. S15. Saliency maps for CDR3β sequences, one example for each epitope species in VDJdbαβ-large dataset (1/2).
Each plot consists of a sequence logo and a heatmap of for CDR3 sequences with the most common length specific to an epitope. The height of a letter in a sequence logo corresponds to that amino acids frequency at that position, and the the background color of the letter shows the average saliency for the amino acid at that position. The heatmap shows the saliency values for each CDR3 sequence individually. The sequences are clustered by the similarity of their saliency values, as illustrated by the dendogram on its left side.

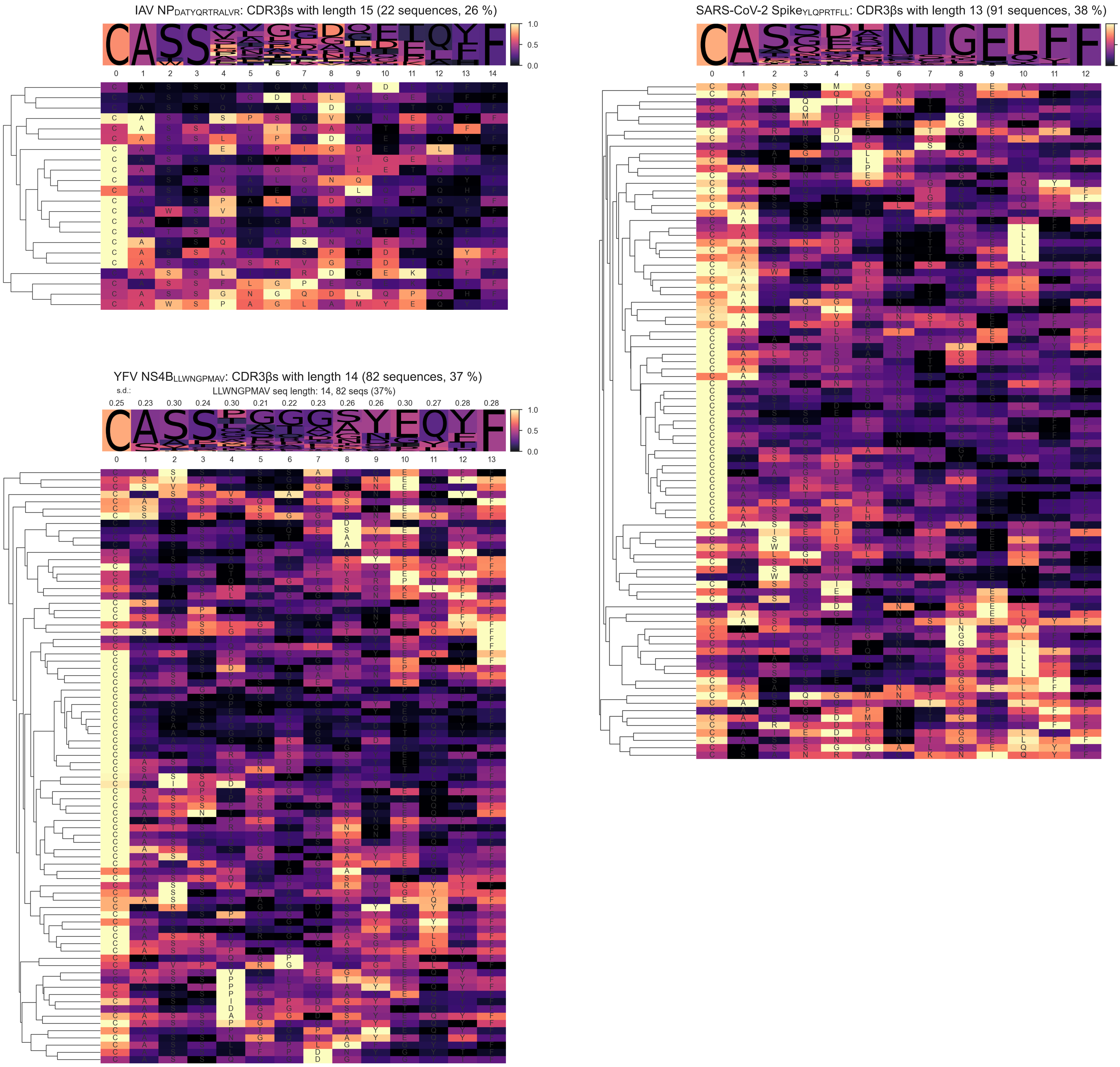

Supplementary Fig. S16. Saliency maps for CDR3β sequences, one example for each epitope species in VDJdbαβ-large dataset (2/2).
Each plot consists of a sequence logo and a heatmap of for CDR3 sequences with the most common length specific to an epitope. The height of a letter in a sequence logo corresponds to that amino acids frequency at that position, and the the background color of the letter shows the average saliency for the amino acid at that position. The heatmap shows the saliency values for each CDR3 sequence individually. The sequences are clustered by the similarity of their saliency values, as illustrated by the dendogram on its left side.

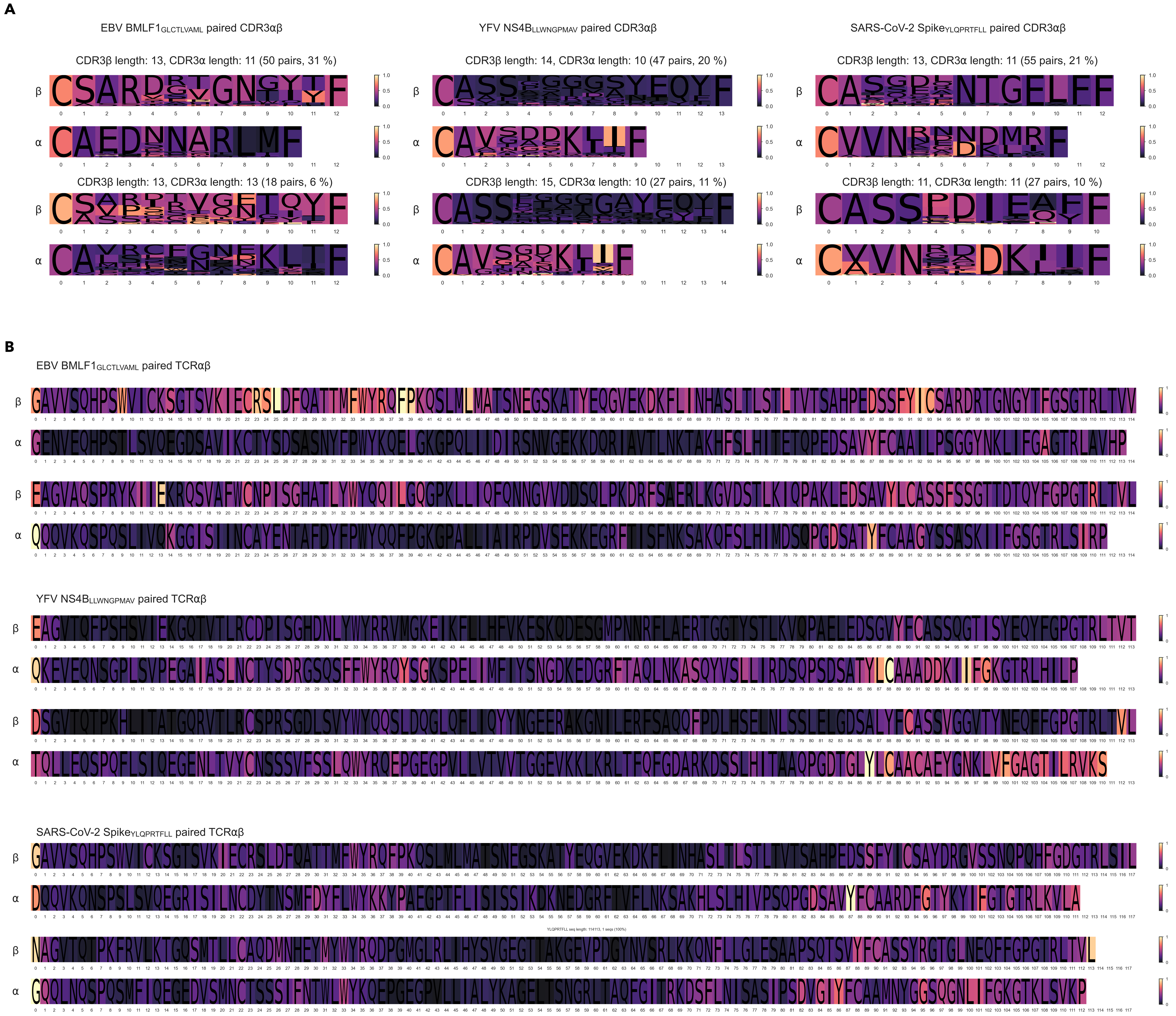

Supplementary Fig. S16. Saliency maps for paired TCRαβ sequences from VDJdbαβ-large dataset, a few examples of TCRs specific to EBV epitope $\text{BMLF1}_{\text{GLCTLVAML}}$, YFV epitope $\text{NS4B}_{\text{LLWNGPMAV}}$, or SARS-CoV-2 epitope $\text{Spike}_{\text{YLQPRTFLL}}$. TCRs specific to $\text{BMLF1}_{\text{GLCTLVAML}}$ epitope have on average higher saliency values for the β-chain, TCRs specific to $\text{NS4B}_{\text{LLWNGPMAV}}$ for the α-chain and with $\text{Spike}_{\text{YLQPRTFLL}}$ the saliency values are quite similar to both chains (see Supplementary Table S7).

(A) Paired CDR3αβ sequences with the two most common lengths specific to EBV epitope $\text{BMLF1}_{\text{GLCTLVAML}}$, YFV epitope $\text{NS4B}_{\text{LLWNGPMAV}}$, or SARS-CoV-2 epitope $\text{Spike}_{\text{YLQPRTFLL}}$. The height of a letter in a sequence logo corresponds to that amino acids frequency at that position, and the background color of the letter shows the average saliency for the amino acid at that position.

(B) Examples of paired TCRαβ sequences.

Supplementary Table S1. Three datasets of epitope-specific TCR-data collected from VDJdb.The datasets contain epitope-specific TCRs for Cytomegalovirus (CMV), Dengue virus types 1, 2 and 3 (DENV1, DENV2, DENV3-4), Epstein-Barr virus (EBV), Hepatitis C virus (HCV), Human immunodeficiency virus type 1 (HIV-1), Influenza A virus (IAV), Severe acute respiratory syndrome coronavirus 2 (SARS-CoV-2), and Yellow Fever virus (YFV), as well as human stromal antigen 2 (BST2), insulin like growth factor 2 mRNA binding protein 2 (IGF2BP2), melanoma antigen (MLANA), and transketolase (TKT). VDJdbβ-large and VDJdbβ-small were collected in January 2021 and VDJdbαβ-large in September 2021 which explains why some of the SARS-CoV-2 epitopes are only present in VDJdbαβ-large.

#	Epitope Species	Epitope Gene	Epitope	MHC chain 1	MHC chain 2	VDJdbβ -large	VDJdbβ -small	VDJdbαβ -large
1	CMV	IE1	KLGGALQAK	HLA-A*03	B2M	12693		13664
2	CMV	IE2	NEGVKAAW	HLA-B*44	B2M	118	62	
3	CMV	pp50	VTEHDTLLY	HLA-A*01	B2M	202		
4	CMV	pp65	IPSINVHHY	HLA-B*35	B2M	92	58	
5	CMV	pp65	NLVPMVATV	HLA-A*02	B2M	4488	244	175
6	CMV	pp65	TPRVTGGGAM	HLA-B*07	B2M	197	122	
7	CMV	pp65	LLQTGIHVRVSQPSL	HLA-DRA1*01	HLA-DRB1*15	304		
8	CMV	pp65	MLNIPSINV	HLA-A*02	B2M	73		
9	CMV	pp65	YSEHPTFTSQY	HLA-A*01	B2M	52		
10	DENV1	NS3	GTSGSPIVNR	HLA-A*11	B2M	165	59	
11	DENV2	NS3	GTSGSPIIDK	HLA-A*11	B2M	60		
12	DENV3-4	NS3	GTSGSPIINR	HLA-A*11	B2M	158	46	
13	EBV	BMLF1	GLCTLVAML	HLA-A*02	B2M	969	159	279
14	EBV	BRLF1	YVLDHLIVV	HLA-A*02	B2M	79	51	
15	EBV	BZLF1	RAKFKQLL	HLA-B*08	B2M	842	151	1212
16	EBV	EBNA3A	RLRAEAQVK	HLA-A*03	B2M	410		422
17	EBV	EBNA4	AVFDRKSDAK	HLA-A*11	B2M	1642		1723
18	EBV	EBNA4	IVTDFSVIK	HLA-A*11	B2M	550		713
19	HCV	NS3	ATDALMTGY	HLA-A*01	B2M	169	139	
20	HCV	NS3	CINGVCWTV	HLA-A*02	B2M	131		76
21	HCV	NS3	KLVALGINAV	HLA-A*02	B2M	65	65	
22	HCV	NS5B	ARMILMTHF	HLA-B*27	B2M	66		
23	HIV-1	Gag	EIYKRWII	HLA-B*08	B2M	148	60	
24	HIV-1	Gag	FRDYVDRFYKTLRAEQASQE	HLA-DRA*01	HLA-DRB1*01,07,11,15, HLA-DRB5*01	367	95	
25	HIV-1	Gag	GPGHKARVL	HLA-B*07	B2M	66	53	
26	HIV-1	Gag	KAFSPEVIPMF	HLA-B*57	B2M	175	104	
27	HIV-1	Gag	KRWIILGLNK	HLA-B*27	B2M	320	141	
28	HIV-1	Gag	KRWIIMGLNK	HLA-B*27	B2M	66		
29	HIV-1	Gag	SLYNTVATL	HLA-A*02	B2M	57		
30	HIV-1	Gag	TPQDLNTML	HLA-B*42,81	B2M	101	40	
31	HIV-1	Nef	FLKEKGGL	HLA-B*08	B2M	144	78	
32	HIV-1	Nef	TPGPGVRYPL	HLA-B*07,42	B2M	67		
33	HIV-1	Pol	ISPRTLNAW	HLA-B*57	B2M	54		
34	HIV-1	Vif	HPKVSSEVHI	HLA-B*42	B2M	54		
35	HIV-1	Vpr	FPRPWLHGL	HLA-B*42	B2M	83		
36	HTLV-1	Tax	SFHSLHLLF	HLA-A*24	B2M	132	45	
37	Human	BST2	LLLGIGILV	HLA-A*02	B2M	233		
38	Human	IGF2BP2	NLSALGIFST	HLA-A*03	B2M	111		
39	Human	MLANA	ELAGIGILTV	HLA-A*02	B2M	1305		388
40	Human	SEC24A	FLYNLLTRV	HLA-A*02	B2M	61		
41	Human	TKT	AMFWSVPTV	HLA-A*02	B2M	82		
42	IAV	HA	PKYVKQNTLKLAT	HLA-DRA*01	HLA-DRB1*01,04	388	69	59
43	IAV	M1	GILGFVFTL	HLA-A*02	B2M	3430	160	1815
44	IAV	M1	GLIYNRMGAVTTEV	HLA-DRA*01	HLA-DRB1*01	121		
45	IAV	M1	QARQMVQAMRTIGTHP	HLA-DRA*01	HLA-DRB1*01	124		
46	IAV	M1	SGPLKAEIAQRLED	HLA-DRA*01	HLA-DRB1*01	64		
47	IAV	NP	DATYQRTRALVR	HLA-A*68	B2M	102		92
48	IAV	NP	DPFRLLQNSQVFS	HLA-DRA*01	HLA-DRB1*01	104		
49	IAV	NP	LPRRSGAAGA	HLA-B*07	B2M	159		
50	YFV	NS4B	LLWNGPMAV	HLA-A*02	B2M	409		239
51	SARS-CoV-2	Spike	YLQPRTFLL	HLA-A*02	B2M	315		261
52	SARS-CoV-2	Spike	LTDEMIAQY	HLA-A*01	B2M			122
53	SARS-CoV-2	Spike	NQKLIANQF	HLA-B*15	B2M			71
54	SARS-CoV-2	NSP3	TTDPSFLGRY	HLA-A*01	B2M			243
55	SARS-CoV-2	Nucleocapsid	SPRWYFYYL	HLA-B*07	B2M			75
					TOTAL epitope-TCR pairs:	32367	2001	21629
					TOTAL unique TCRs	30503	1977	20200

Supplementary Table S2. Method comparison. Mean AUROC and AP scores for TCRconv, TCRGP, TCRdist, SETE, DeepTCR and ERGO-II from stratified 10-fold cross-validation. Mean AUROC and AP scores are macro averages over all epitopes. Standard deviation is given over all folds and over all epitopes (Epit.), showing that with all methods variation between folds is smaller than variation between different epitopes. With TCRconv we have used protBERT embeddings for CDR3 + full context, meaning that the embedding is first computed for the complete TCR (as defined by the CDR3 and V- and J-genes), but only the parts of the embeddings correponding to the CDR3 are used with the predictor. For TCRGP, DeepTCR and TCRdist the results were computed with models using only CDR3βs or additionally other components of TCRβs. With these methods accuracies were higher when additional components were used. All result figures present the more accurate version of each method.

Method	TCRβ parts	VDJdbβ-small Mean AUROC	VDJdbβ-small Standard deviation Folds	VDJdbβ-small Standard deviation Epit.	VDJdbβ-small Mean AP	VDJdbβ-small Standard deviation Folds	VDJdbβ-small Standard deviation Epit.	VDJdbβ-large Mean AUROC	VDJdbβ-large Standard deviation Folds	VDJdbβ-large Standard deviation Epit.	VDJdbβ-large Mean AP	VDJdbβ-large Standard deviation Folds	VDJdbβ-large Standard deviation Epit.
TCRconv	CDR3 + full context	0.853	0.028	0.064	**0.595**	0.054	0.164	**0.770**	0.010	0.108	**0.283**	0.016	0.205
TCRGP	CDR3	0.801	0.029	0.074	0.451	0.045	0.195	0.675	0.010	0.117	0.168	0.011	0.177
	all CDRs	**0.860**	0.026	0.062	0.544	0.054	0.175	0.728	0.014	0.106	0.202	0.014	0.192
DeepTCR	CDR3	0.752	0.019	0.088	0.356	0.024	0.188	0.705	0.007	0.108	0.173	0.009	0.173
	CDR3+V	0.797	0.022	0.072	0.438	0.031	0.174	0.747	0.005	0.101	0.213	0.012	0.194
SETE	CDR3	0.679	0.022	0.099	0.265	0.027	0.175	0.569	0.012	0.086	0.049	0.003	0.091
TCRdist	CDR3	0.702	0.027	0.082	0.334	0.028	0.150	0.641	0.008	0.116	0.131	0.008	0.139
	all CDRs	0.770	0.029	0.105	0.432	0.034	0.190	0.704	0.008	0.125	0.183	0.009	0.169
ERGO-II	CDR3+V	0.761	0.022	0.099	0.248	0.026	0.130	0.673	0.028	0.102	0.053	0.005	0.091

$$\text{standard deviation (epit.)} = \sqrt{\frac{\sum_{e=1}^{N_e}(S_e - S)^2}{N_e - 1}}$$

$$\text{standard deviation (folds)} = \sqrt{\frac{\sum_{f=1}^{N_f}(S_f - S)^2}{N_f - 1}}$$

$$S_e = \sum_{f=1}^{N_f}\frac{S_{e,f}}{N_f}, \quad S_f = \sum_{e=1}^{N_e}\frac{S_{e,f}}{N_e}, \quad S = \sum_{e=1}^{N_e}\sum_{f=1}^{N_f}\frac{S_{e,f}}{N_e N_f}$$

N_e is the number of epitopes (21 in VDJdbβ-small, 51 in VDJdbβ-large),
N_f is the number of folds (10),
$S_{e,f}$ is the mean score (AUROC or AP) for epitope e in fold f
S_e is the mean score for epitope e over all folds,
S_f is the mean score for fold f over all epitopes, and
S is the mean score over all epitopes and folds.

Supplementary Table S3. Overview of ImmuneCODE MIRA data used for training TCRconv classifiers for SARS-CoV-2 specific epitopes. Spike refers to surface glycoprotein, membrane to membrane glycoprotein, and nucleocapsid to nucleocapsid phosphoprotein. The peptide groups are ordered by the start point of their genomic location (Loc). The coloring of the genomic regions matches Supplementary Fig. 8.

#	ORF	Loc	Peptides	TCRs
1	ORF1ab, *spike*	533	AELEGIQY, *TLADAGFIK, LADAGFIKQY, ADAGFIKQY*	108
2	ORF1ab	587	VPHVGEIPVAY, GEIPVAYRKVLL	709
3	ORF1ab	1391	SEVGPEHSLAEY	318
4	ORF1ab	2024	TSDLATNNLVVMAY	68
5	ORF1ab	2468	APKEIIFL, KEIIFLEGETL	2444
6	ORF1ab	3137	KPLEFGATSAAL	716
**7**	ORF1ab	3707	LLSAGIFGA	52
**8**	ORF1ab,*ORF3a*	3875	AEIPKEEVKPF, *SASKIITLK, ASKIITLKK*	170
9	ORF1ab	4211	ALRKVPTDNYITTY, KVPTDNYITTY	643
10	ORF1ab	4346	SNEKQEILGTVSW, ILGTVSWNL	632
**11**	ORF1ab	5171	HTTDPSFLGRY	10134
12	ORF1ab	5834	SEYKGPITDVFY, ITDVFYKENSY	376
13	ORF1ab	6074	FADDLNQLTGY	89
14	ORF1ab	6521	ITEEVGHTDLMAAY	226
15	ORF1ab	7253	AYILFTRFFYV	243
16	ORF1ab	7415	YIFFASFYY	457
**17**	ORF1ab	7952	QLMCQPILL, QLMCQPILLL	1062
18	ORF1ab	8060	SSTFNVPMEKLK	82
19	ORF1ab	8111	AEAELAKNVSL, AELAKNVSLDNVL	1866
20	ORF1ab	8660	HTDFSSEIIGY	56
21	ORF1ab	8915	FLPRVFSAV	1520
22	ORF1ab	9602	TFYLTNDVSFL	53
23	ORF1ab	9812	FLLNKEMYL	85
24	ORF1ab	10472	FLNGSCGSV	4506
25	ORF1ab	10664	VLAWLYAAV	263
26	ORF1ab	10709	FLNRFTTTL	235
27	ORF1ab	11168	FLCLFLLPSL, FLLPSLATV	339
28	ORF1ab	11228	MPASWVMRI	815
29	ORF1ab	11492	NYSGVVTTVMF	72
30	ORF1ab	11684	TLGVYDYLV, GVYDYLVST	104
31	ORF1ab	11921	KLWAQCVQL	741
32	ORF1ab	12530	FTYASALWEI	69
33	ORF1ab	12599	SEISMDNSPNL	208
**34**	ORF1ab	13058	TVLSFCAFA, VLSFCAFAV	1095
35	ORF1ab	13805	YTMADLVYA, TMADLVYAL	373
36	ORF1ab	14159	LLMPILTLT	122
37	ORF1ab	14360	ILHCANFNV	353
38	ORF1ab	14402	FPPTSFGPL	686
39	ORF1ab	14441	FVDGVPFVV	5402
40	ORF1ab	14789	ISDYDYYRY	71
41	ORF1ab	14840	RQLLFVVEV	1393
**42**	ORF1ab	15437	MVMCGGSLYV, VMCGGSLYV	879
**43**	ORF1ab	15641	NRDVDTDFVNEFY, DTDFVNEFYAY	326
44	ORF1ab	16358	LVLSVNPYV	110
45	ORF1ab	16673	KLSYGIATV	4607
46	ORF1ab	16862	VVYRGTTTY	222
47	ORF1ab	16889	KLNVGDYFV	372
48	ORF1ab	16952	TLVPQEHYV	274
49	ORF1ab	17024	SSNVANYQK	108
50	ORF1ab	17579	IVDTVSALV	124
51	ORF1ab	17810	ILGLPTQTV	451
52	ORF1ab	18011	IPRRNVATL	72
53	ORF1ab	18590	VLWAHGFEL	1392
54	ORF1ab	18998	ALLADKFPV	50
55	ORF1ab	19520	YLDAYNMMI	500
56	ORF1ab	19538	MMISAGFSL	51
57	ORF1ab, [illegible]	19895	APAHISTI, *LIVNSVLLFL, SVLLFLAFV, LLFLAFVVFL*	1555
58	ORF1ab	20510	LLLDDFVEI, LLDDFVEII	2031
59	ORF1ab	20681	GVAMPNLYK	51
60	ORF1ab	20816	YLNTLTLAV	828
61	ORF1ab	20987	TLIGDCATV	856
62	spike	21632	LPPAYTNSF	145
63	spike	21668	VYYPDKVFR, YPDKVFRSS, YPDKVFRSSV, KVFRSSVLH	121
64	spike	21710	STQDLFLPFF, TQDLFLPFF	155
**65**	spike	21725	FLPFFSNVT, LPFFSNVTW, LPFFSNVTWF, PFFSNVTWF, FFSNVTWFH, SNVTWFHAI	455
66	spike	21809	VLPFNDGVY, VLPFNDGVYF, LPFNDGVYF, LPFNDGVYFA, DGVYFASTEK, GVYFASTEK	1467
**67**	spike	21887	TLDSKTQSL	171
68	spike	21965	FCNDPFLGVY, CNDPFLGVY, CNDPFLGVYY	224
69	spike, *membrane*	21986	GVYYHKNNK, YYHKNNKSW, *VPLHGTIL*	62
70	spike	22010	KSWMESEFRV, SWMESEFRVY, WMESEFRVY	179
71	spike	22037	VYSSANNCTF, SSANNCTFEY	240
72	spike	22058	CTFEYVSQPF, FEYVSQPFL, EYVSQPFLM	427
73	spike	22184	TPINLVRDL	268
74	spike	22229	LEPLVDLPI	536
75	spike	22244	DLPIGINITR, LPIGINITRF, INITRFQTL	99
**76**	spike	22355	YYVGYLQPRTF, YLQPRTFL, YLQPRTFLL	1217
77	spike	22451	SETKCTLKSF, CTLKSFTVEK, TLKSFTVEK	150
78	spike	22520	VQPTESIVRF, QPTESIVRF, TESIVRFPNI, VRFPNITNL, RFPNITNLCPF, FPNITNLCPF	1460
79	spike	22574	FGEVFNATRF, GEVFNATRF, FNATRFASVY, NATRFASVY	836
80	spike	22631	RISNCVADY	67
81	spike	22655	YSVLYNSASF, SVLYNSASF, LYNSASFSTF, NSASFSTFK	62
82	spike	22718	CFTNVYADSF, FTNVYADSF, FTNVYADSFV, KLNDLCFTNV, LNDLCFTNVY, NVYADSFVIR, VYADSFVIR	484
83	spike	22784	APGQTGKIA, GQTGKIADY, KIADYNYKL, QTGKIADYNY, RQIAPGQTGK	118
84	spike	22832	KLPDDFTGCV	1381
**85**	spike	22880	NLDSKVGGNY	54
**86**	spike	22904	NYLYRLFRK, NYNYLYRLF	536
87	spike	22946	FERDISTEI, FERDISTEIY, KPFERDISTEI	223
88	spike	23027	YFPLQSYGF	434
89	spike	23051	FQPTNGVGY	62
90	spike	23072	GYQPYRVVVL, PYRVVVLSF, QPYRVVVL, QPYRVVVLSF	263
91	spike	23309	EILDITPCSF	278
92	spike	23372	TSNQVAVLY	87
93	spike	23465	STGSNVFQTR, TGSNVFQTR, VYSTGSNVF	348
94	spike	23513	GAEHVNNSY, IGAEHVNNSY	83
95	spike	23552	IGAGICASY, IPIGAGICASY	136
96	spike	23579	QTNSPRRAR, SPRRARSVA, SYQTQTNSPR, TQTNSPRRAR	115
97	spike	23615	ASQSIIAYTM, RSVASQSII, SIIAYTMSL, SQSIIAYTM, VASQSIIAY	721
98	spike	23678	AIPTNFTISV, AYSNNSIAIPTNF, IPTNFTISV, NSIAIPTNF	771
99	spike	23714	FTISVTTEIL	204
100	spike	23759	KTSVDCTMYI	174
**101**	spike	23816	LLLQYGSFC, LLQYGSFCT	93
102	spike	24023	LLFNKVTLA	101
103	spike	24140	LLTDEMIAQY, LTDEMIAQY, LTDEMIAQYT, VLPPLLTDEMIAQY	1080
104	spike	24200	GTITSGWTF	72
105	spike	24242	IPFAMQMAY, LQIPFAMQM	83
**106**	spike	24317	NQKLIANQF	82
**107**	spike	24485	SVLNDILSR, VLNDILSRL	55
**108**	spike	24509	RLDKVEAEV	50
109	spike	24521	AEVQIDRLI, AEVQIDRLIT, VEAEVQIDRL, VQIDRLITGR	246
110	spike	24548	GRLQSLQTY, LITGRLQSL, RLQSLQTYV	464
111	spike	24608	AEIRASANL, AEIRASANLA, ASANLAATK	211
112	spike	24701	HLMSFPQSA, YHLMSFPQSA	106
113	spike	24716	FPQSAPHGV, FPQSAPHGVVF	222
114	spike	24728	APHGVVFL, APHGVVFLHV, GVVFLHVTY, VVFLHVTYV	403
115	spike	24752	HVTYVPAQEK, TYVPAQEKNF, VTYVPAQEK	73
116	spike	24857	GTHWFVTQR	111
117	spike	24968	TVYDPLQPELDSFK	53
118	spike	25103	KEIDRLNEV	80
119	spike	25136	NLNESLIDL	203
120	spike	25178	QYIKWPWYI, YEQYIKWPW, YEQYIKWPWY	1751
121	spike	25220	FIAGLIAIV	323
122	spike	25268	CMTSCCSCLK, MTSCCSCLK	159
123	spike	25343	SEPVLKGVKL	149
124	ORF3a	25393	DLFMRIFTI, MDLFMRIFTI	114
125	ORF3a	25408	RIFTIGTVTLK	140
126	ORF3a	25474	FVRATATIPI	53
127	ORF3a	25495	IPIQASLPF	124
128	ORF3a	25531	IVGVALLAVF, VGVALLAVF	54
**129**	ORF3a	25579	ITLKKRWQL, LKKRWQLAL, TLKKRWQLA, TLKKRWQLAL	143
130	ORF3a	25606	ALSKGVHFV	397
131	ORF3a	25627	FVCNLLLLFV, LLFVTVYSHL, TVYSHLLLV	1495
132	ORF3a	25690	FLQSINFVR, FLQSINFVRI, FLYLYALVYF, GLEAPFLYLY, INFVRIIMR, LQSINFVRI, LQSINFVRII, QSINFVRII, SINFVRIIMR, VYFLQSINF, VYFLQSINFV, YFLQSINFVR, YLYALVYFL	3298
133	ORF3a	25801	LLYDANYFL, LLYDANYFLC, LYDANYFLCW, NPLLYDANY, PLLYDANYFL, YDANYFLCW	1039
134	ORF3a	25930	SEHDYQIGGYTEKW, YQIGGYTEK, YQIGGYTEKW	3447
135	ORF3a	25996	SYFTSDYYQL, VLHSYFTSDY, YFTSDYYQLY	1174
136	ORF3a	26050	GVEHVTFFIY, HVTFFIYNK, STDTGVEHVTFFIY, VEHVTFFIY	304
137	ORF3a	26113	EEHVQIHTI	178
138	envelope	26260	SEETGTLIV	63
139	envelope	26392	SLVKPSFYV	128
140	membrane	26538	GTITVEELK	62
141	membrane	26553	EELKKLLEQW, KLLEQWNLV, QWNLVIGFLF	187
142	membrane	26613	WICLLQFAY	595
143	membrane	26625	FAYANRNRF, LQFAYANRNR, YANRNRFLY	416
144	membrane	26652	FLYIIKLIFL, FLYIIKLVFL, LYIIKLIFL, LYIIKLIFLW, RFLYIIKLIF, YIIKLIFLW, YIIKLIFLWL	950
145	membrane	26679	FLWLLWPVT, FLWLLWPVTL, LWLLWPVTL, LWPVTLACF, TLACFVLAAV, WLLWPVTLA, [illegible]	5441
146	membrane	26763	AIAMACLVGL, IAMACLVGLM	112
147	membrane	26802	FIASFRLFA, SYFIASFRLF, YFIASFRLF, YFIASFRLFA	372
148	membrane	26844	MWSFNPETNI, SFNPETNIL, SMWSFNPET	412
149	membrane	26928	SELVIGAVI, SELVIGAVIL	1564
150	membrane	26964	HLRIAGHHL, RIAGHHLGR	101
151	membrane	27027	ATSRTLSYY, ATSRTLSYYK, TSRTLSYYK, TVATSRTLSY	334
152	membrane	27069	ASQRVAGDSGFAAY, DSGFAAYSR, VAGDSGFAAY	57
153	ORF6	27208	HLVDFQVTI	174
154	ORF6	27226	VTIAEILLI	130
155	ORF6	27241	FKVSIWNLDY, ILLIIMRTFK, IMRTFKVSI, KVSIWNLDY, KVSIWNLDYI, LIIMRTFKV, RTFKVSIWNL, SIWNLDYII, TFKVSIWNL, VSIWNLDYII	727
156	ORF7a	27418	CELYHYQECV, ITLATCELY, LITLATCELY, TLATCELYHY	609
157	ORF7a	27454	QECVRGTTVL	203
158	ORF7a	27511	YEGNSPFHPL	101
159	ORF7a	27523	HPLADNKFAL, SPFHPLADNKFAL	252
160	ORF7a	27580	CPDGVKHVY, DGVKHVYQL, FAFACPDGVKHVY	187
161	ORF7a	27625	RARSVSPKL, SVSPKLFIR	136
162	ORF7a	27670	ELYSPIFLI, LYSPIFLIV, QELYSPIFL, VQELYSPIF, VQELYSPIFL	2332
163	ORF7a	27715	VFITLCFTL, VFITLCFTLK	138
164	ORF7b	27756	AFLLFLVLI, FLAFLLFLV, FYLCFLAFL, FYLCFLAFLL, IDFYLCFLAF, IELSLIDFYL, LIDFYLCFL, LLFLVLIML, MIELSLIDFY, SLIDFYLCFL, YLCFLAFLL	16778
165	ORF7b	27822	IMLIIFWFSL, MLIIFWFSL	1452
166	ORF8	27984	VDDPCPIHFY, VVDDPCPIHFY, YVVDDPCPI	356
167	ORF8	28104	IQYIDIGNY	241
168	ORF8	28122	GNYTVSCLPF, NYTVSCLPF, YTVSCLPFTI	360
169	ORF8	28206	DFLEYHDVR, EDFLEYHDVR, LEYHDVRVV, LEYHDVRVVL, YEDFLEYHDVRVVL	1749
170	nucleocapsid	28466	FPRGQGVPI, KFPRGQGVPI	62
171	nucleocapsid	28496	NSSPDDQIGY, NTNSSPDDQIGYY, SSPDDQIGY, SSPDDQIGYY	166
172	nucleocapsid	28529	YYRRATRRIR	84
173	nucleocapsid	28550	RIRGGDGKM, RIRGGDGKMK	107
174	nucleocapsid	28583	LSPRWYFYY, SPRWYFYYL	3084
175	nucleocapsid	28673	ALNTPKDHI, ATEGALNTPK	509
176	nucleocapsid	28718	NPANNAAIV, NPANNAAIVL, RNPANNAAIV	357
177	nucleocapsid	28745	GTTLPKGFY, LQLPQGTTL, QLPQGTTLPK, TTLPKGFYA, VLQLPQGTTL	129
178	nucleocapsid	28916	ALALLLLDR, GDAALALLLL, LALLLLDRL, LLLDRLNQL, LLLLDRLNQ, LLLLDRLNQL	870
179	nucleocapsid	28988	QQQGQTVTK, QQQQGQTVTK	80
180	nucleocapsid	29069	KAYNVTQAF	1450
181	nucleocapsid	29138	ELIRQGTDY, QELIRQGTDY, QELIRQGTDYKHW	166
182	nucleocapsid	29186	APSASAFFGM, AQFAPSASA, ASAFFGMSR, SASAFFGMSR	494
183	nucleocapsid	29219	GMSRIGMEV, SRIGMEVTPSGTW	55
184	nucleocapsid	29234	GMEVTPSGTWL, MEVTPSGTWL, TPSGTWLTY, VTPSGTWLTY	2003
185	nucleocapsid	29348	AYKTFPPTEPK, KTFPPTEPK	1645
186	nucleocapsid	29456	LPAADLDDF	138
187	ORF10	29558	AFPFTIYSL, GYINVFAFPF, INVFAFPFTI, MGYINVFAF, NVFAFPFTI, NVFAFPFTIY, YINVFAFPF	5160
188	ORF10	29630	AQVDVVNFNL, NYIAQVDVV	119

Supplementary Table S4. Healthy control and ImmuneCODE repertoire data used in the analysis for T-cell dynamics during COVID-19 (Fig. 2a). The controls consist of the first 72 TCR repertoires from healthy (CMV-) subjects in cohort 1 in the study of Emerson et al. that had over 250 000 TCRs, number of templates reported, and where the subject is known to be at least 18 years old (which is the age of the youngest subject in the ImmuneCODE data used here). From ImmuneCODE 493 repertoires with over 250 000 TCRs and "Days from diagnosis to sample" reported were selected from four separate datasets.

Cohort type	Cohort name	Institution	Study description	Mean age and s.d. (years)	Number of samples	Samples with ≥ 250000 TCRs and Days from diagnosis to sample reported
Healthy control	Emerson	Fred Hutchinson Cancer Research Center	Human peripheral blood samples were obtained from the institution's Research Cell Bank biorepository of healthy bone marrow donors. Donors underwent CMV serostatus testing at the time the samples were taken	36.1 ± 8.9		72
COVID-19	ImmuneRACE (ADI)	Adaptive Biotechnologies	Whole blood samples were collected from subjects from 24 geographic areas in the US with active infection, in convalescent phase, or exposed to SARS-CoV-2	42.6 ± 11.9	123	118
COVID-19	ISB	Institute for Systems Biology	Whole blood samples collected under the INCOVE project at Providence St. Joseph Health (Seattle, WA). Subjects were enrolled during the active phase and monitored through disease.	66.1 ± 16.7	157	48
COVID-19	DLS	Discovery Life Sciences	Whole blood samples collected during routine care in acucte and convalescdent phases procured through Discovery Life Sciences (Huntsville, AL).	64.1 ± 18.5	431	216
COVID-19	H12O	Hospital Universitario 12 de Octubre	Whole blood samples were collected at the Hospital Universitario 12 de Octubre (Madrid, Spain) during the active or convalescent phase.	60.5 ± 19.1	612	111
					TOTAL:	110 + 493

Supplementary Table S5. Significance of case-control and age effects on frequency of virus specific T-cells. Linear regression analysis was performed to assess if COVID patients have significantly higher frequency of virus specific T-cells than healthy control subjects, and if frequencies are positively correlated with subjects' age (see Methods). **(A)** The Benjamini-Hochberg adjusted p-values representing the significance of $b_{cc} > 0$. **(B)** The Benjamini-Hochberg adjusted p-values representing the significance of $b_{age} > 0$. One-tailed t-test was used for computing the p-values and the multiple testing adjustments are done for each virus (column) separately. Adjusted p-values smaller than 0.1 are bolded.

A

Time interval	SARS-CoV-2	CMV	IAV	EBV	HCV
0-2	0.3443	0.9181	1.0000	1.0000	1.0000
3-7	**0.0087**	0.8991	1.0000	0.7298	0.9982
8-14	**0.0596**	1.0000	1.0000	0.7324	1.0000
15-28	**0.0104**	0.8760	1.0000	0.7707	1.0000
29-42	0.2457	1.0000	1.0000	0.6010	1.0000
43	0.1790	0.9486	1.0000	0.7892	1.0000

B

Time interval	SARS-CoV-2	CMV	IAV	EBV	HCV
0-2	**0.0033**	0.0970	0.1009	0.4553	0.4698
3-7	0.2982	**0.0261**	0.1380	**0.0632**	0.4236
8-14	0.1846	0.1186	0.6596	0.6491	0.4310
15-28	**0.0166**	**0.0029**	0.1296	**0.0460**	0.2211
29-42	**0.0000**	0.1024	0.6254	0.3875	0.1598
43	**0.0157**	0.0354	0.6434	0.3731	0.4855

Supplementary Table S6. Embedding comparison. Mean AUROC and AP scores from stratified 10-fold cross-validation with TCRconv on VDJdbβ-small and VDJdbβ-large datasets using different embeddings.

Embedding	VDJdbβ-small		VDJdbβ-large	
	Mean AUROC	Mean AP	Mean AUROC	Mean AP
protBERT	0.853	**0.595**	**0.770**	**0.283**
Finetuned protBERT	0.848	0.575	0.740	0.260
Masked ELMo	0.847	0.571	0.763	0.278
ELMo	0.838	0.539	0.761	0.261
One-hot TCR	**0.855**	0.567	0.751	0.192
One-hot CDR3	0.797	0.481	0.710	0.190

Supplementary Table S7. Average position-wise saliency values for TCRs specific to each epitope in VDJdbαβ-large dataset. Values are given separately for α- and β-chains for the CDR3 region and the complete TCR, defined by the V- and J-genes and CDR3.

Epitope species	Epitope gene	Epitope	CDR3		TCR	
			α-chain	β-chain	α-chain	β-chain
CMV	IE1	KLGGALQAK	0.303	**0.377**	0.146	**0.197**
CMV	pp65	NLVPMVATV	0.329	**0.343**	0.161	**0.190**
EBV	BMLF1	GLCTLVAML	0.266	**0.449**	0.133	**0.234**
EBV	BZLF1	RAKFKQLL	0.291	**0.344**	0.149	**0.183**
EBV	EBNA3A	RLRAEAQVK	0.297	**0.353**	0.132	**0.197**
EBV	EBNA4	AVFDRKSDAK	0.308	**0.391**	0.140	**0.193**
EBV	EBNA4	IVTDFSVIK	0.321	**0.352**	0.168	**0.191**
HCV	NS3	CINGVCWTV	**0.410**	0.286	**0.297**	0.168
Human	MLANA	ELAGIGILTV	**0.398**	0.309	**0.224**	0.162
IAV	HA	PKYVKQNTLKLAT	0.227	**0.463**	0.116	**0.189**
IAV	M	GILGFVFTL	0.262	**0.470**	0.121	**0.200**
IAV	NP	DATYQRTRALVR	**0.362**	0.288	**0.216**	0.171
YFV	NS4B	LLWNGPMAV	**0.402**	0.224	**0.231**	0.168
SARS-CoV-2	Spike	YLQPRTFLL	**0.361**	0.350	0.175	**0.181**
SARS-CoV-2	Spike	LTDEMIAQY	0.252	**0.422**	0.110	**0.183**
SARS-CoV-2	Spike	NQKLIANQF	**0.338**	0.291	**0.176**	0.172
SARS-CoV-2	NSP3	TTDPSFLGRY	0.339	**0.389**	0.154	**0.212**
SARS-CoV-2	Nucleocapsid	SPRWYFYYL	**0.353**	0.286	**0.215**	0.160
